# The rice blast fungus MoRgs1 functioning in cAMP signaling and pathogenicity is regulated by casein kinase MoCk2 phosphorylation and modulated by membrane protein MoEmc2

**DOI:** 10.1371/journal.ppat.1009657

**Published:** 2021-06-16

**Authors:** Rui Yu, Xuetong Shen, Muxing Liu, Xinyu Liu, Ziyi Yin, Xiao Li, Wanzhen Feng, Jiexiong Hu, Haifeng Zhang, Xiaobo Zheng, Ping Wang, Zhengguang Zhang

**Affiliations:** 1 Department of Plant Pathology, College of Plant Protection, Nanjing Agricultural University, and Key Laboratory of Integrated Management of Crop Diseases and Pests, Ministry of Education, Nanjing, China, The Key Laboratory of Plant Immunity, Nanjing Agricultural University, Nanjing, China; 2 Departments of Microbiology, Immunology, and Parasitology, and Pediatrics, Louisiana State University Health Sciences Center, New Orleans, Louisiana, United States of America; Sainsbury Laboratory, UNITED KINGDOM

## Abstract

GTP-binding protein (G-protein) and regulator of G-protein signaling (RGS) mediated signal transduction are critical in the growth and virulence of the rice blast pathogen *Magnaporthe oryzae*. We have previously reported that there are eight RGS and RGS-like proteins named MoRgs1 to MoRgs8 playing distinct and shared regulatory functions in *M*. *oryzae* and that MoRgs1 has a more prominent role compared to others in the fungus. To further explore the unique regulatory mechanism of MoRgs1, we screened a *M*. *oryzae* cDNA library for genes encoding MoRgs1-interacting proteins and identified MoCkb2, one of the two regulatory subunits of the casein kinase (CK) 2 MoCk2. We found that MoCkb2 and the sole catalytic subunit MoCka1 are required for the phosphorylation of MoRgs1 at the plasma membrane (PM) and late endosome (LE). We further found that an endoplasmic reticulum (ER) membrane protein complex (EMC) subunit, MoEmc2, modulates the phosphorylation of MoRgs1 by MoCk2. Interestingly, this phosphorylation is also essential for the GTPase-activating protein (GAP) function of MoRgs1. The balance among MoRgs1, MoCk2, and MoEmc2 ensures normal operation of the G-protein MoMagA-cAMP signaling required for appressorium formation and pathogenicity of the fungus. This has been the first report that an EMC subunit is directly linked to G-protein signaling through modulation of an RGS-casein kinase interaction.

## Author summary

G-proteins play a significant role in signal perception and transduction during pathogen and host interactions. In the rice blast fungus *M*. *oryzae*, previous studies demonstrated that G-protein/cAMP signaling are important for appressorium formation and pathogenicity. One of the eight regulator of G-protein signaling (RGS) and RGS-like proteins, MoRgs1, targets G-protein MoMagA to regulate cAMP levels and growth and virulence of the fungus; however, how MoRgs1 exhibits this function and its own regulation indifferent from other RGS and RGS-like proteins are not clear. We here demonstrated that MoRgs1 is subject to regulation by the casein kinase 2 MoCk2 through protein phosphorylation, and this regulation is also essential for the GTPase-activating protein (GAP) function of MoRgs1. We also showed that the endoplasmic reticulum (ER) membrane complex (EMC) subunit MoEmc2 modulates MoCk2-mediated MoRgs1 phosphorylation. Balanced interactions among MoRgs1, MoEmc2, and MoCk2 ensure normal appressorium formation and pathogenicity of *M*. *oryzae*.

## Introduction

Heterotrimeric G-protein signaling plays a fundamental role in regulating various cellular developmental processes in eukaryotic organisms. The largest family of membrane receptors, G-protein-coupled receptors (GPCRs), sense extracellular stimuli by activating G-proteins, consisting of three canonical subunits Gα, Gβ, and Gγ, and downstream effector molecules that modify cellular behaviors [1–3]. In a conserved fashion, activated GPCRs induce conformational changes in the GDP-bound Gαβγ heterotrimer leading to the dissociation of GTP-bound Gα from the Gβγ heterodimer [4]. GTP-bound Gα can then convey signals to adenylyl cyclases (ACs) that modulate the levels of cyclic adenosine monophosphate (cAMP) and cAMP-dependent protein kinase A (PKA) pathways [5–7]. The Gβ subunit was also shown to be involved in both cAMP/PKA and Pmk1 MAPK pathways important in appressorium formation and virulence of the fungus [8–11]. The regulator of G-protein signaling proteins (RGS) is known to serve as GTPase-activating proteins (GAP) for various Gα subunits of heterotrimeric G proteins [12–14]. In addition, some RGS proteins contain additional domains such as the GoLoco motif that inhibits GDP dissociation [15].

Appressoria are the unique infectious structures produced by the rice blast fungus *Magnaporthe oryzae* during infection. With its robust turgor pressure, mature appressoria effectively penetrate the host cell allowing rapid invasion and colocalization [16–19]. G-protein-mediated cAMP signaling has been demonstrated to be crucial for various aspects of signal transduction and substrate transport during appressorial biogenesis in *M*. *oryzae* [20–23]. Previous studies have demonstrated that Pth11 (a non-canonical GPCR), MoMagA (Gα), and MoRgs1 (RGS) mediate the perception of hydrophobic and hard surface cues leading to the activation of the cAMP-PKA pathway and normal appressorial formation [20, 24–26].

MoRgs1 is one of the eight RGS and RGS-like proteins whose function is linked to multiple Gαs in *M*. *oryzae* [21, 22, 26–29]. MoRgs1 have the N-terminal DEP domains involved in G-protein signaling and a dynamic tubulo-vesicular localization [21, 30, 31]. Despite its important function in *M*. *oryzae*, regulation of MoRgs1 remains not clear. Previous studies in the budding yeast *Saccharomyces cerevisiae* suggested that the RGS protein Sst2 is regulated by a phosphorylation feedback loop involving the MAP kinase Fus3 in response to pheromone stimulation [32, 33]. A phosphoproteome study indicated that MoRgs1 could be subject to phosphorylation regulation [34]; however, further studies were not done until today.

The CK2 protein kinase is an evolutionally conserved serine/threonine kinase in eukaryotes functioning in many cellular processes [35, 36]. A typical Ck2 holoenzyme is tetrameric, with two catalytic and two regulatory subunits [37]. CK2 was found to function as a member of diverse kinases that regulate GPCRs [38]. In the chestnut blight fungus *Cryphonectria parasitica*, CK2 phosphorylates the phosducin-like protein Bdm-1 that is required for the Gβ subunit protein stability and virulence [39]. A previous study identified one catalytic subunit Ckα and two regulatory subunits Ckβ1 and Ckβ2 from *M*. *oryzae*, which were named MoCka1, MoCkb1, and MoCkb2, respectively [40]. MoCka1 encodes an essential function, whereas MoCkb1 and MoCkb2 have a role in regulating the pathogenicity of *M*. *oryzae* [37, 40].

The ER membrane protein complex (EMC) is conserved proteins identified in mammals and yeasts [41]. Yeast has eight EMC proteins that aggregate around the ER compartment [41–43]. Emc2, Emc8, and Emc9 do not contain ER targeting signals, thus constituting a part of the EMC cytoplasmic interface [44]. Interestingly, Emc2 contains the conserved tetratricopeptide repeat (TPR) domains for protein-protein interactions [45–48]. EMC functions typically as an insertase and/or membrane protein chaperone required for the biogenesis of dominant proteins in lipid homeostasis and signal transduction [44, 49, 50]. To further explore the regulatory mechanism of MoRgs1, we here demonstrated that MoRgs1 is subject to MoCk2 regulation through protein phosphorylation, and this regulation is required for MoRgs1 GAP function. We also identified that MoCk2-dependent MoRgs1 phosphorylation is modulated by MoEmc2. These concordant cellular processes ensure a critical role of G-protein/cAMP-PKA signaling in regulating appressorium formation and pathogenicity of *M*. *oryzae*.

## Results

### MoRgs1 is phosphorylated during the developmental and invasive stages of *M*. *oryzae*

To determine whether MoRgs1 is subject to phosphorylation regulation, we performed an *in vivo* phosphorylation assay using Mn^2+^-Phos-tag gel electrophoresis analysis. We generated a MoRgs1-GFP fusion construct and introduced it into the Δ*Morgs1* strain. Cell extracts were obtained from mycelial, conidial, and appressorial stages treated either with or without a phosphatase or a phosphatase inhibitor. The results showed that a specific mobility shift pattern of MoRgs1-GFP consistent with that MoRgs1 could be phosphorylated in *M*. *oryzae* (S1 Fig).

### MoCk2 is a protein kinase that phosphorylates MoRgs1

To identify proteins that bind to and potentially phosphorylate MoRgs1, we performed a yeast two-hybrid (Y2H) screen to identify MoRgs1-interacting proteins. We constructed a MoRgs1-BD bait vector and screened a cDNA activator library of *M*. *oryzae*. Following gene sequencing analysis of several binding partners, we identified MoCkb2 (encoded by loci MGG_05651) (S1 Text).

**Fig 1:**
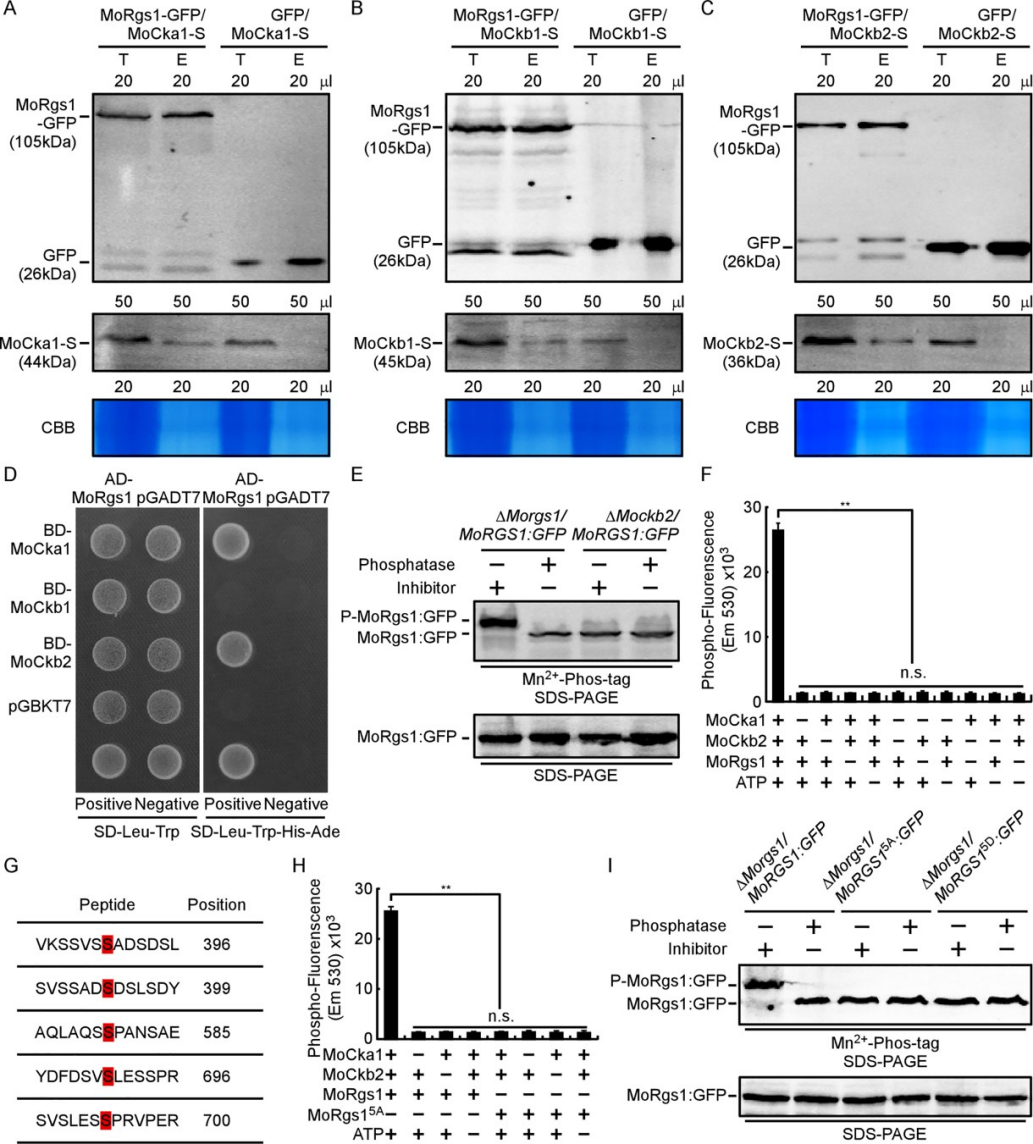
MoCk2 functions as a kinase for MoRgs1 phosphorylation. (A-C) Co-IP assays among all three subunits of MoCk2 holoenzyme (MoCka1-S, MoCkb1-S, and MoCkb2-S) and MoRgs1-GFP. Empty GFP transformants introduced by all three subunits of the Ck2 holoenzyme (MoCka1-S, MoCkb1-S, and MoCkb2-S) were the controls. Total proteins were extracted individually as the total proteins, then eluted from the anti-GFP agarose beads and analyzed by Western blot with corresponding antibodies. CBB: Coomassie brilliant blue staining indicates loading controls. T: Total protein. E: Elution. (D) Y2H assays among all three subunits of MoCk2 holoenzyme (BD-MoCka1, BD-MoCkb1, and BD-MoCkb2) and AD-MoRgs1. pGADT7 and pGBKT7 fused with specific genes were co-introduced into the yeast AH109 strain. Transformants were isolated on SD-Leu-Trp plate and screened by SD-Ade-His-Leu-Trp plates for 5 d. The pair of plasmids pGBKT7-Lam and pGADT7-RECT were used as a negative control. (E) Phosphorylation analysis of MoRgs1 *in vivo* by Mn^2+^-Phos-tag gel. MoRgs1-GFP proteins treated with phosphatase and phosphatase inhibitors were detected by the GFP antibody and shifted by Mn^2+^-Phos-tag SDS-PAGE and normal SDS-PAGE, respectively. (F) Phosphorylation analysis *in vitro* by the fluorescence detection in tube (FDIT) method. Purified proteins of GST-MoCka1, GST-MoCkb2, and His-MoRgs1 were constructed for protein kinase reactions in the presence of ATP. The fluorescence signal was measured in a microplate reader. Asterisks denote statistically significant difference according to ANOVA (**P < 0.01, n = 3). Values are means of three replications and standard deviation (SD). (G) Identification of differentiated phosphorylation sites in the wild type strain Guy11 compared with Δ*Mockb2* strains by LC-MS-MS (Q-E). (H) Phosphorylation analysis by the fluorescence detection in tube (FDIT) method *in vitro*. GST-MoCka1, GST-MoCkb2, His-MoRgs1, and His-MoRgs1^5A^ (S396A, S399A, S585A, S696A, and S700A) were constructed for protein kinase reaction assays in the presence of ATP and a kinase reaction buffer. Fluorescence was measured in a microplate reader. Fluorescence at 590 nm (excited at 530 nm) was measured. Values are means of three replications and SD (**P < 0.01, n = 3). (I) Phosphorylation analysis of MoRgs1 and site-directed mutagenesis MoRgs1^5A^ and MoRgs1^5D^ *in vivo*. Proteins were extracted from corresponding transformants and treated with phosphatase and phosphatase inhibitors, then detected by the GFP antibody and shifted by Mn^2+^-Phos-tag SDS-PAGE and normal SDS-PAGE, respectively. All experiments were conducted with three biological repetitions and three replicates.

As the MoCk2 holoenzyme is composed of MoCka1 and MoCkb1 and MoCkb2, we tested the interactions of all three proteins with MoRgs1 by *in vivo* co-IP and split YFP BiFC assays and by *in vitro* Y2H and GST-pulldown assays. The results showed that MoRgs1 interacts with all three *in vivo* (Figs 1A, 1B, 1C and S2B), but with MoCka1 and MoCkb2 *in vitro* (Figs 1D and S2A). The BiFC assay found that fluorescence appeared at the inner PM and dynamic tubulo-vesicular compartments that are similar to the localization of MoRgs1 [30, 31] (S2B Fig).

Previous studies indicated that both yeast CK2 catalytic subunits are essential and that the regulatory subunit of CK2 is required for modulating substrate specificity and stabilizing against denaturing forces [37, 40, 51]. To examine whether MoCkb1 and MoCkb2 have a role in regulating MoRgs1 through their kinase function, we performed an *in vivo* phosphorylation assay that found MoCkb2, but not MoCkb1, is involved in MoRgs1 phosphorylation (Figs 1E and S3A). To confirm the phosphorylation between MoCk2 and MoRgs1, we performed an *in vitro* fluorescence detection in tube (FDIT) assay that showed MoRgs1 phosphorylation is dependent on both MoCka1 and MoCkb2 (Fig 1F). Individual subunits of MoCk2 or all three together would not support this phosphorylation (S3B Fig). Together, these results indicated that MoCka1 and MoCkb2 are required for phosphorylating MoRgs1. Despite that MoCkb1 interacts with MoRgs1 *in vivo*, it does not seem to be involved in MoRgs1 phosphorylation.

### MoCk2 phosphorylates S396, S399, S585, S696, and S700 residues of MoRgs1

To identify the phosphorylation sites of MoRgs1 by MoCk2, we compared LC-MS/MS analysis data between the wild-type (WT) and Δ*Mockb2* strains and identified five differentiated serine phosphorylation sites, including S396, S399, S585, S696, and S700 (Figs 1G and S4). To test if these five serine residues are MoCk2-dependent phosphorylation sites, we generated all five Serine (S) to Alanine (A) and Aspartic acid (D) site-directed mutagenesis constructs fused with either GFP or GST to mimic the sustainable unphosphorylated (MoRgs1^5A^) and phosphomimetic (MoRgs1^5D^) status. Phosphorylation assays with *in vivo* Mn^2+^-Phos-tag gel analysis and the *in vitro* FDIT method confirmed that all five serine residues of MoRgs1 are the phosphorylation sites by MoCk2 (Fig 1H and 1I).

### MoRgs1 phosphorylation is required for cAMP signaling

**Fig 2:**
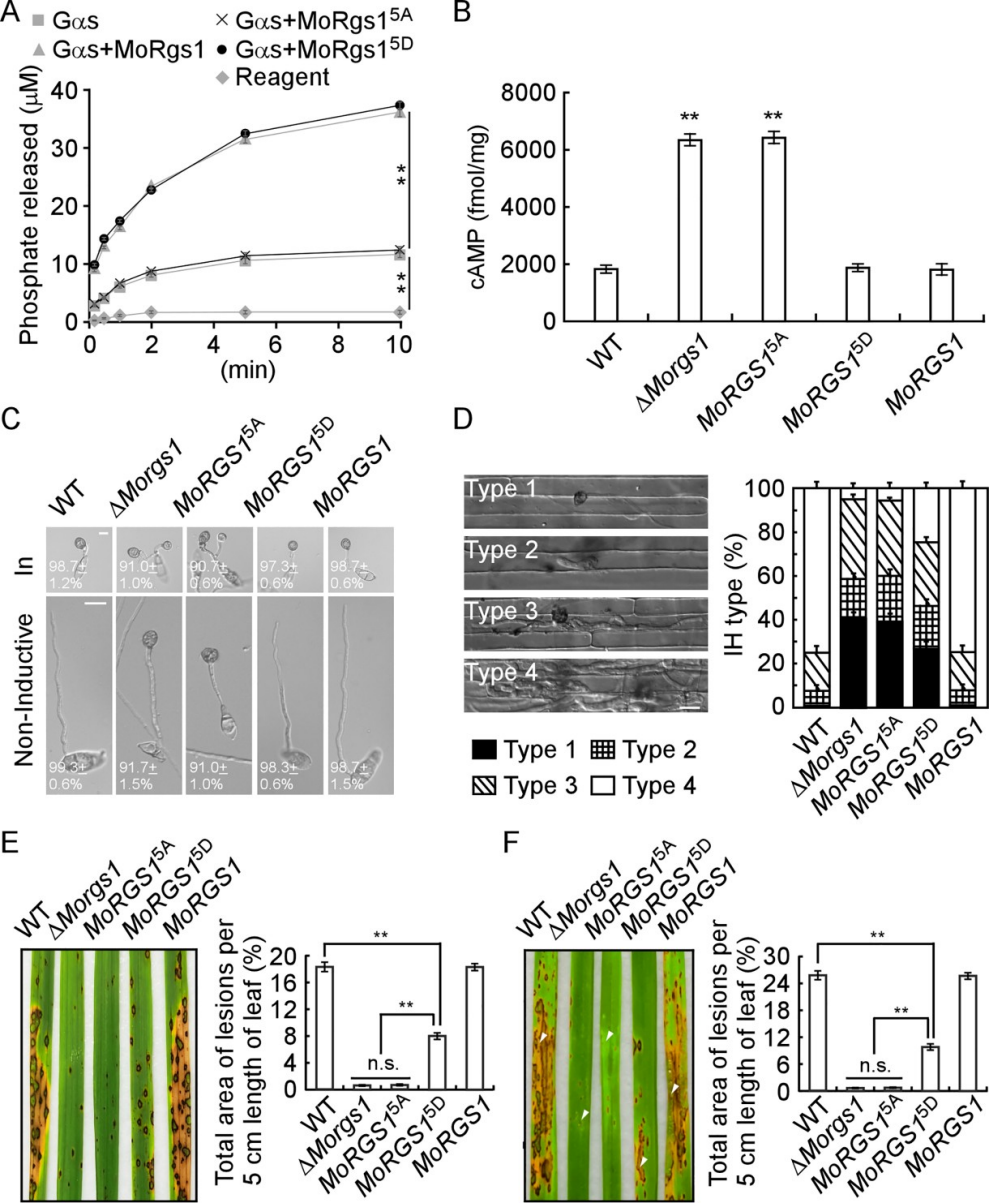
MoRgs1 phosphorylation is required for the appressorium formation and virulence of *M*. *oryzae*. (A) Measurement of MoRgs1 and its site-directed mutagenesis MoRgs1^5A^ and MoRgs1^5D^ accelerating the GTPase (released phosphate by time) rate of Gαs in a single catalytic turnover at room temperature using the GTPase activity kit. Values are means of three replications and SD (**P < 0.01, n = 3). (B) Intracellular cAMP levels in mycelia of indicated strains cultured for 2 d in the complete medium (CM) and quantified by HPLC. Values are means of three replications and SD (**P < 0.01, n = 3). (C) Appressorium formation assay was conducted on non-inductive or inductive (In) surfaces. Conidia from the WT, Δ*Morgs1*, *MoRGS1*^5A^ (Δ*Morgs1/MoRGS1*^5A^), *MoRGS1*^*5D*^ (Δ*Morgs1/MoRGS1*^5D^), and complemented *MoRGS1* (Δ*Morgs1/MoRGS1*) strains were dropped onto corresponding surfaces and photographed by confocal microscopy at 24 h. Values are means of three replications and SD (**P < 0.01, n = 100). (D) Detailed observation with statistical analysis for infectious hyphal growth in rice sheath cells at 24 hpi. Four milliliters of conidial suspension (1 × 10^5^ spores/ml) of each strain were used for the injection. Appressorium penetration sites were observed, and IH were rated from type 1 to type 4 (n = 100). Error bars represent SD from three independent replicates. (E) Rice spraying assays *in vivo* and lesion area statistics. Conidial suspensions (5 × 10^4^ spores/ml) were sprayed onto 2-week-old rice seedlings (CO-39). Diseased rice leaves were photographed and the percentage of per 5 cm length leaf lesion area were analyzed by ImageJ after 7 days of inoculation (**P < 0.01, n = 10). Error bars represent SD from three independent replicates. (F) Rice sheath injecting assays *in vivo* and lesion area statistics. Conidial suspensions (2 × 10^5^ spores/ml) were sprayed onto 4-week-old rice seedlings (CO-39). Diseased rice leaves were photographed, and the percentage of per 5 cm length leaf lesion area was analyzed by ImageJ after 5 days of inoculation (**P < 0.01, n = 10). Error bars represent SD from three independent replicates. White triangles point out the injection sites.

To test the roles of MoRgs1 phosphorylation, we evaluated the *in vitro* GAP function by measuring levels of free phosphate release using an ATPase/GTPase activity assay kit. The mimic sustainable unphosphorylated MoRgs1^5A^ loses its GAP function in comparison to MoRgs1 and mimic phosphomimetic MoRgs1^5D^, indicating MoRgs1 phosphorylation is required for its GAP function (Fig 2A). Considering the GAP function of MoRgs1 is critical for maintaining the intracellular cAMP levels in *M*. *oryzae* [21, 22], we tested the levels in Δ*Morgs1* and site-directed mutagenesis transformants by HPLC. The mimic sustainable unphosphorylated MoRgs1^5A^ transformants have higher cAMP levels than the wild-type strains (Fig 2B), indicating jeopardizing MoRgs1 phosphorylation equals to the loss of the GAP function.

In addition, we tested the interaction among GDP-bound MoMagA with MoRgs1, MoRgs1^5A^, and MoRgs1^5D^ by co-IP assays. The results showed that MoRgs1 and MoRgs1^5A^ interact with MoMagA, but not MoRgs1^5D^ (S5 Fig), which indicated that phosphorylated MoRgs1 would be dismissed from the heterotrimeric G-protein Gα subunit.

### MoRgs1 phosphorylation is required for appressorium formation and pathogenicity

Because MoRgs1 is involved in G-protein cAMP signaling and the Δ*Morgs1* strains maintain normal appressorium on non-inductive surfaces [21, 22, 52], we tested the roles of MoRgs1 phosphorylation in appressorium formation at the hydrophobic and hydrophilic surfaces, respectively. The mass of two dome-shaped appressoria was generated when the phosphorylation of MoRgs1 was compromised (MoRgs1^5A^) at the hydrophobic surface (Fig 2C). In addition, when MoRgs1 losses phosphorylation (MoRgs1^5A^), it promotes appressorium formation at the hydrophilic surface (Fig 2C). Therefore, the phosphorylation of MoRgs1 activated by MoCk2 is important for invasive structure morphogenesis in *M*. *oryzae*.

Because the appressorium functions as an invasive structure, we hypothesized that MoRgs1 phosphorylation plays a significant role in the pathogenicity of *M*. *oryzae*. To determine its function, we observed the invasive hyphae (IH) growth into epidermal cells of rice leaf sheath by confocal microscopy (Fig 2D). We also evaluated virulence by leaf spraying and *in vivo* sheath injection assays (Fig 2E and 2F). The results indicated that the balance of MoRgs1 phosphorylation is critical for tbe pathogenicity of *M*. *oryzae*. The sustainable unphosphorylated MoRgs1 showed virulence defects that were similar to the Δ*Morgs1* strain. Even though the sustainable phosphomimetic MoRgs1 partially suppresses the defect, it still showed a significant difference from that of the wild type and complemented strains.

In addition, phenotypes growth, biomass, conidiation, and penetration were assessed for MoRgs1 and the site-directed mutagenesis strains (S2 Text). Consistent with the pathogenicity results, both sustainable unphosphorylated and phosphomimetic MoRgs1 exhibit significant defects compared to WT and the complemented strains. All of the above data showed that the phosphorylation of MoRgs1 has a significant role in G-protein signaling required for appressorium formation and pathogenicity.

### MoEmc2 interacts with MoRgs1 and all three subunits of MoCk2

To further characterize the modulation of MoRgs1 phosphorylation by MoCk2 in G-protein/cAMP signaling, we identified a tetratricopeptide repeat (TPR) domain-containing protein, MoEmc2, encoded by the gene locus MGG_07480 (S1 Text) from a Y2H screening. MoEmc2 shares a high amino acid sequence homology to yeast Emc2, an evolutionarily conserved ER membrane protein complex subunit (S6A Fig). To further validate this conclusion, we conducted a yeast complementation assay based on that the yeast Δ*Scemc2* strain has a growth defect at the restrictive high temperature [49, 53]. We introduced *MoEMC2* into the yeast Δ*Scemc2* strain using the expression vector pYES2 and the results showed that MoEmc2 did restore the growth to the yeast Δ*Scemc2* strain (S6B Fig).

**Fig 3:**
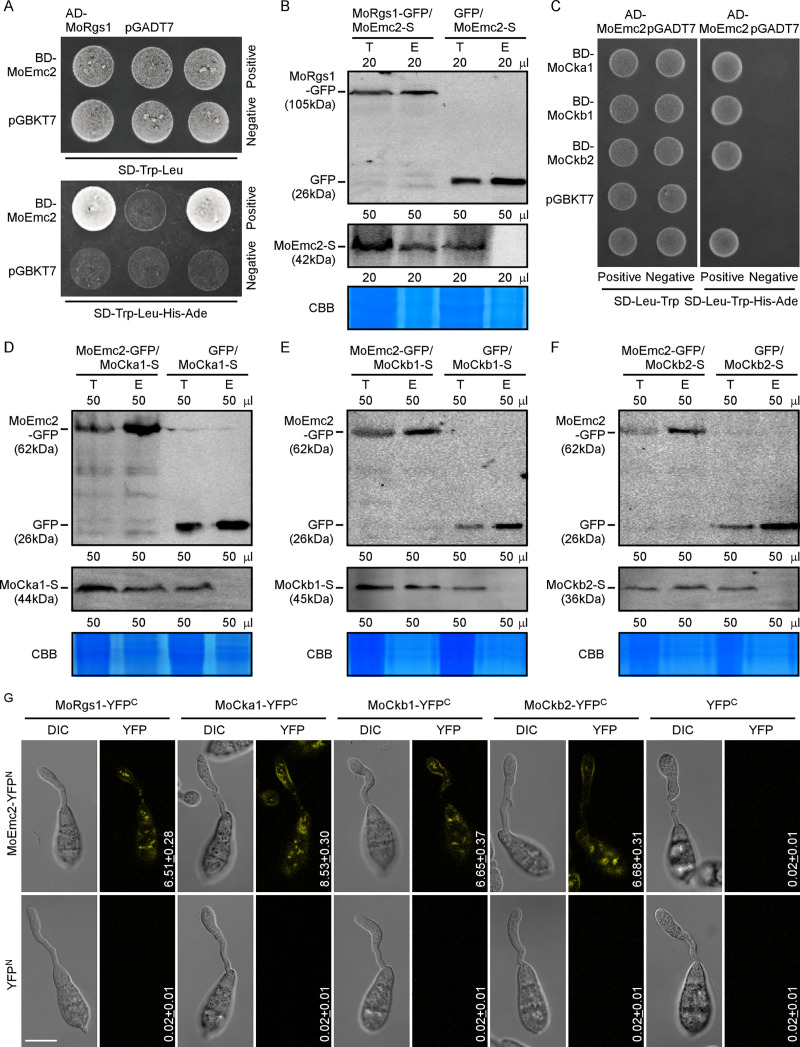
MoEmc2 interacts with MoRgs1 and MoCk2. (A) Y2H assays for examining interactions between AD-MoRgs1 and BD-MoEmc2. Yeast co-transformants expressing the bait and prey constructs were isolated on SD-Leu-Trp plate for 3 days and screened by culturing on SD-Ade-His-Leu-Trp plates for 5 days. (B) Co-IP assays for the interaction between MoRgs1-GFP with MoEmc2-S. GFP transformants were introduced into MoEmc2-S as a control. Total proteins were extracted individually as the total proteins, eluted from the anti-GFP agarose beads, and analyzed by Western blot with the anti-GFP or anti-S antibodies. CBB: Coomassie brilliant blue staining indicates loading controls. T: Total protein. E: Elution. (C) Y2H assays for examining the interaction among all three subunits of MoCk2 holoenzyme (BD-MoCka1, BD-MoCkb1, and BD-MoCkb2) with AD-MoEmc2. (D-F) Co-IP assays for interactions among all three subunits of MoCk2 holoenzyme (MoCka1-S, MoCkb1-S, and MoCkb2-S) with MoEmc2-GFP, respectively. GFP transformants were introduced into all three subunits of the MoCk2 holoenzyme (MoCka1-S, MoCkb1-S, and MoCkb2-S) as controls. CBB: Coomassie brilliant blue staining indicates loading controls. T: Total protein, E: Elution. (G) BiFC assays for interactions among all three subunits of MoCk2 holoenzyme (MoCka1-YFP^C^, MoCkb1-YFP^C^, and MoCkb2-YFP^C^) and MoRgs1-YFP^C^ with MoEmc2-YFP^N^. Empty YFP^C^ and empty YFP^N^ constructs were used as a negative control. Co-transformants were observed at the germ tube hooking stage (3 h after conidial suspension dropped onto the hydrophobic surface) with LSM (Zeiss LSM710 laser scanning microscope, 63 × oil, Bar = 10 μm). The mean and SD of fluorescence intensity were denoted that calculated over 50 cells. All experiments were conducted with three biological repetitions and three replicates.

To validate the interaction between MoRgs1 and MoEmc2, we performed Y2H, co-IP, and BiFC assays. All data showed that MoRgs1 interacts with MoEmc2 *in vivo* and *in vitro* (Fig 3A, 3B and 3G). Interestingly, confocal fluorescence in the BiFC assay is similar to that for MoRgs1-MoCk2 interactions, nearby the PM and dynamic tubulo-vesicular compartments at the germ tube hooking stage (Fig 3G). This suggests that MoEmc2 shares a similar localization as MoRgs1-MoCk2 interactions. We again tested the interactions among all three subunits of MoCk2 and MoEmc2 by Y2H, co-IP, and BiFC assays. Unlike MoRgs1, MoEmc2 directly interacts with all three subunits MoCka1, MoCkb1, and MoCkb2 *in vivo* and *in vitro* (Fig 3C–3G). In addition, confocal fluorescence in the BiFC assay confirmed that MoEmc2 interacts with MoCk2 holoenzyme subunits at the inner PM and tubule-vesicular compartments (Fig 3G).

### MoEmc2 interacts with MoRgs1 at the N terminus

To explore the MoEmc2-MoRgs1 interaction, we used SMART (http://smart.embl-heidelberg.de/) to predict protein interactive domains and motifs. MoEmc2 contains at least one centrally located TPR domain (160-193aa), whereas MoRgs1 contains two DEP domains at the N terminal and one RGS domain at the C terminal. The DEP domain at the N terminal is suggested to primarily function by targeting the C terminal RGS core domain to the punctate vesicular structures [22, 31]. We divided the sequence of MoEmc2 amino acids into three regions, including the N terminal domain (NTD), the TPR regions (TPR), and the C terminal domain (CTD). MoRgs1 was divided into two regions with the DEP containing the N-terminal domain (NTD) and the RGS domain-containing the C-terminal domain (CTD). Y2H assays indicated interactions between the NTDs of MoEmc2 and MoRgs1 (S7 Fig).

### MoEmc2 is required for MoCk2-dependent MoRgs1 phosphorylation

To further test the roles of MoEmc2 in MoCk2-dependent MoRgs1 phosphorylation, we performed targeted gene replacement and complement assays [54]. Putative hygromycin-resistant transformants were screened and mutants confirmed by Southern blot analysis (S8 Fig). Two independent Δ*Moemc2* transformants showed the same defective phenotypes. In addition, the complemented strains were also phenotyped to be similar to the WT strains. Only one mutant and one complemented strain were used for further analysis.

**Fig 4:**
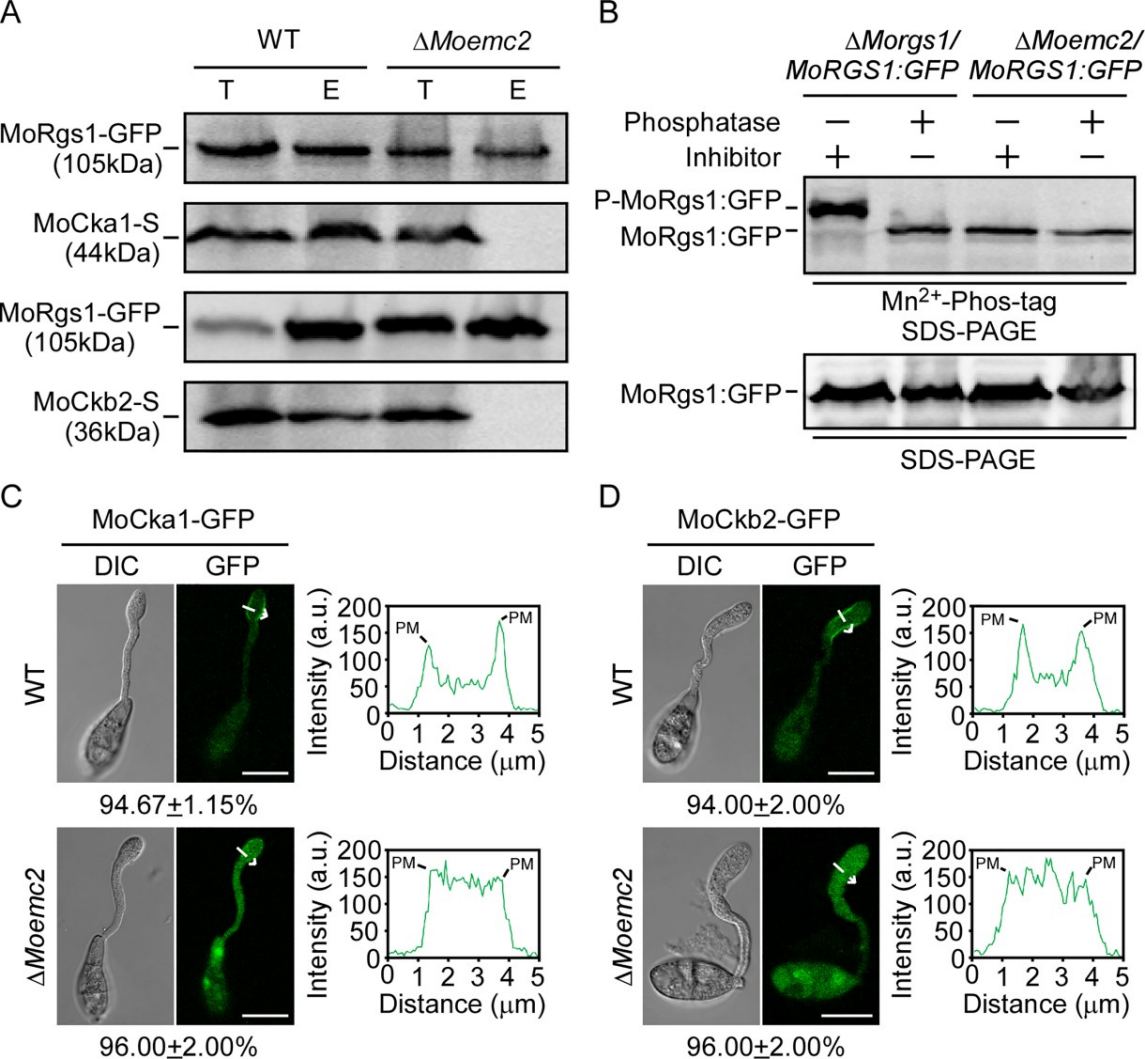
MoEmc2 is required for MoCk2 function and proper subcellular localization. (A) Co-IP assays for interactions between MoRgs1-GFP and MoCka1-S and MoCkb2-S in the wild type (WT) and mutant Δ*Moemc2* strains. Total proteins were extracted and eluted from the anti-GFP agarose beads before being analyzed by Western blot with corresponding antibodies. T: Total protein E: Elution. (B) Phosphorylation analysis of MoRgs1 in the WT and Δ*Moemc2* strains *in vivo* by Mn^2+^-Phos-tag gel electrophoresis. MoRgs1-GFP fusion proteins treated with phosphatase and phosphatase inhibitors were detected by the GFP antibody and shifted by Mn^2+^-Phos-tag SDS-PAGE and normal SDS-PAGE, respectively. (C and D) Fluorescence RFP labeled MoCka1 and MoCkb2 fusion constructs were introduced into the WT and Δ*Moemc2* strains, respectively. Transformants were observed and photographed by LSM (Zeiss LSM710 laser scanning microscope, 63 × oil, Bar = 10 μm) at the germ tube hooking stage. Insets highlight areas analyzed by line-scan (length = 5 μm). The percentage of a pattern showed in the image was calculated by observation for 50 germinated conidia that were randomly chosen. All experiments were conducted with three biological repetitions and three replicates.

Because MoEmc2 contains the TPR domain for protein-protein interaction, and it interacts with all three subunits of the MoCk2 holoenzyme and MoRgs1, we hypothesized that it might act as a scaffold in MoRgs1 phosphorylation. The interactions among MoCk2 holoenzyme subunits with MoRgs1 were tested in the wild type and Δ*Moemc2* strains by co-IP assays (Figs 4A and S9C). As expected, the loss of MoEmc2 hinders the interactions among MoCk2 holoenzyme subunits with MoRgs1. Based on the defect in interaction, we further tested the effect of MoEmc2 in modulating MoCk2-dependent MoRgs1 phosphorylation. *In vivo* phosphorylation assay was performed. Similar to MoCk2-mediated MoRgs1 phosphorylation, MoEmc2 is required for MoRgs1 phosphorylation (Fig 4B). We also tested the phosphorylation sites in the Δ*Moemc2* mutant by LC-MS/MS and found that all five serine residues are unphosphorylated (S4G Fig). The results indicated that MoEmc2 is also required for MoRgs1 phosphorylation.

Considering MoEmc2 is a membrane protein, we hypothesized it might modulate the subcellular localization of MoCk2 holoenzyme subunits or/and MoRgs1. We observed the subcellular localization of MoCk2 and MoRgs1 by laser scanning microscopy (LSM) in the wild type and Δ*Moemc2* strains at the germ tube hooking stage. The subcellular localization of all three subunits of MoCk2 at tubule-vesicular compartments and inner PM is modulated by MoEmc2, while it does not affect MoRgs1 (Figs 4C, 4D, S9A and S9B). Together, these results indicated that MoEmc2 modulates the subcellular localization of MoCk2 to govern MoRgs1 phosphorylation.

### MoEmc2 is required for the development, differentiation, and full virulence of *M*. *oryzae*

As MoRgs1 phosphorylation is required for the development and pathogenicity of *M*. *oryzae*, we further explored the roles of MoEmc2 in development and differentiation. We tested the growth, conidiation, biomass, penetration, and the collapsed appressorium rate. Significant defects were observed between the Δ*Moemc2* and the wild type and complemented strains (S3 Text). All of the results indicated that MoEmc2 is required for the development and reproduction of *M*. *oryzae*.

During the pathogen-host interaction, *M*. *oryzae* perceives the hard, hydrophobic surface of rice leaf and generates an infectious structure appressorium. G-protein/cAMP signaling usually promotes the germination of conidia, and the Pmk1 MAPK pathway is also required for appressorium maturation and IH growth [8, 16, 26, 29, 55]. Because MoEmc2 is involved in G-protein signaling, we determined the roles of MoEmc2 in the pathogen-host infection by rice spraying, *in vivo* sheath penetration, and *in vitro* assays (S10A–S10F Fig). Consistent with a role in modulating the development, MoEmc2 is also critical for the virulence and invasive growth of *M*. *oryzae*.

As appressorium is important for invasion, we tested the appressorium formation rate at continuous time points and calculated it at the hydrophobic and hydrophilic surfaces at 24 h. The results indicated that MoEmc2 not only regulates conidia germination at the early stage but also dominates appressorium maturation (S10G and S10H Fig). In addition, similar to MoRgs1, MoEmc2 is required for appressorium formation at the hydrophilic surface, even though with a lower rate (S10I Fig). As for morphology, MoEmc2 is required for normal appressorium development (S10K Fig). However, when we tested the cellular cAMP levels in the transformants, MoEmc2 and MoRgs1 showed differential effects (S10J Fig). Unlike the negative regulation in G-protein/cAMP signaling of MoRgs1, MoEmc2 exhibits a positive effect similar to the adenylyl cyclase MoMac1. The reasons for this discrepancy remain to be explored.

### Activating MoRgs1 phosphorylation partially inhibits deficiency of the Δ*Moemc2* mutant

**Fig 5:**
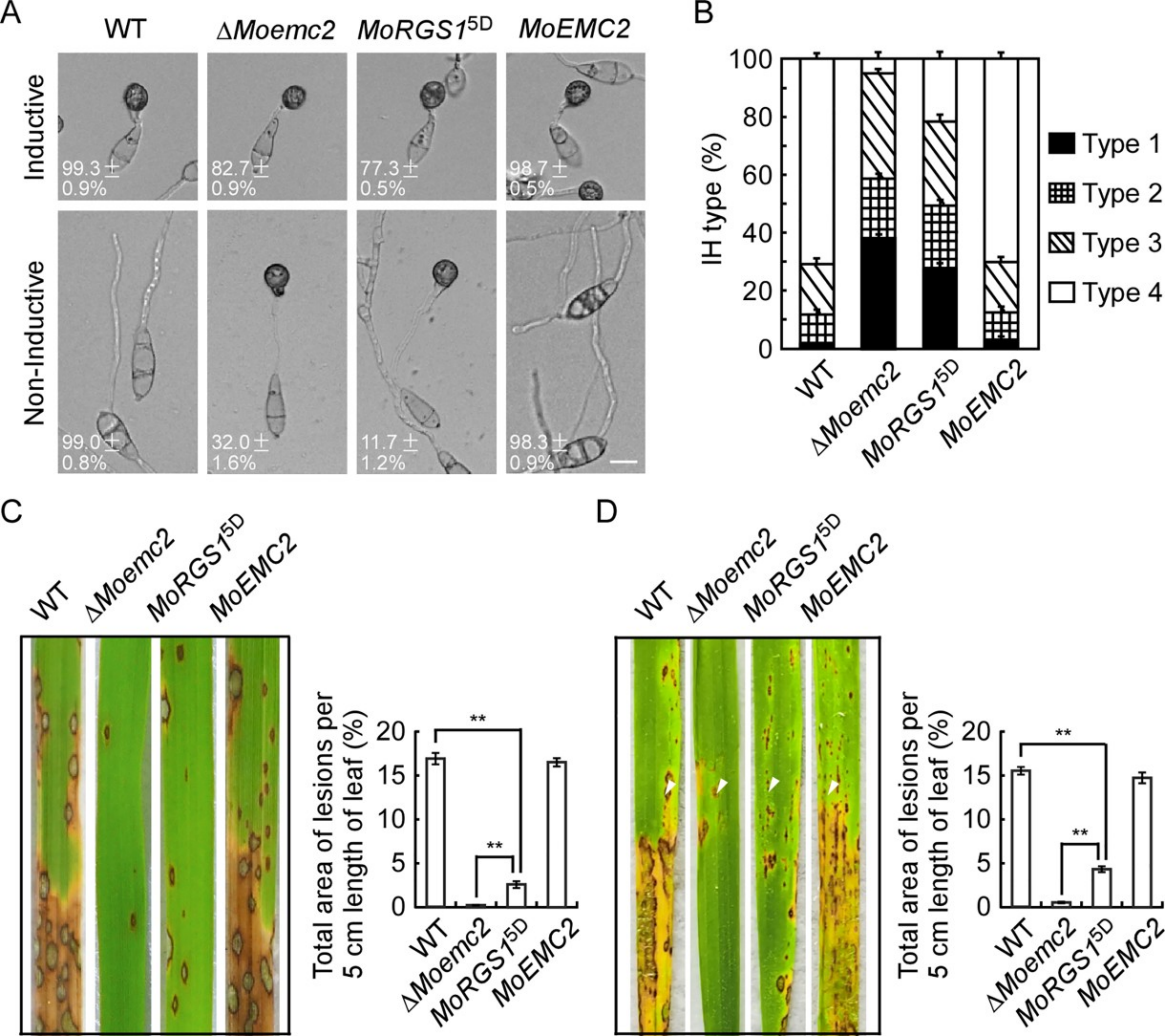
Activating MoRgs1 phosphorylation inhibits appressorium formation and pathogenicity deficiency in Δ*Moemc2* mutant strains. (A) Appressorium formation assays and statistics analysis. Conidia of WT, Δ*Moemc2*, *MoRGS1*^5D^ (Δ*Moemc2/MoRGS1*^5D^), and complemented *MoEMC2* (Δ*Moemc2/MoEMC2*) strains were dropped on the hydrophobic and hydrophilic surfaces. The morphology of appressorium formation was observed at 24 h and photographed by confocal microscopy. Bar = 10 μm. Conidia (n = 100) were observed and calculated with three replicates, and values are means of three replications and SD. (B) Detailed observation with statistical analysis for infectious hyphal growth in rice sheath cells at 24 hpi. Four milliliters of conidial suspension (1 × 10^5^ spores/ml) of each strain were used for the injection. Appressorium penetration sites were observed, and IH were rated from type 1 to type 4 (n = 100). Error bars represent SD from three independent replicates. (C) Rice spraying assays and lesion area statistics. Conidial suspensions (5 × 10^4^ spores/ml) were sprayed onto 2-week-old rice seedlings (CO-39). Diseased rice leaves were photographed, and percentages of per 5 cm length leaf lesion area were analyzed by ImageJ after 7 days of inoculation (**P < 0.01, n = 10). (D) Rice sheath injecting assays *in vivo* and lesion area statistics. Conidial suspensions (2 × 10^5^ spores/ml) were sprayed onto 4-week-old rice seedlings (CO-39). Diseased rice leaves were photographed, and percentages of per 5 cm length leaf lesion area were analyzed by ImageJ after 5 days of inoculation (**P < 0.01, n = 10). White triangles point out the injection sites.

Because sustainable activated MoRgs1 phosphorylation could partly inhibit the phenotype defect in *M*. *oryzae*, we tested the effects of phosphomimetic MoRgs1^5D^ in the Δ*Moemc2* mutant. First, appressorium formation was observed and calculated at the hydrophobic and hydrophilic surfaces. The results showed that activating phosphorylation of MoRgs1 strengthens the defect of appressorium formation rate at the hydrophobic surface but partly suppresses the appressorium formation at the hydrophilic surface, suggesting the disturbance of MoRgs1 phosphorylation is, to some extent, responsible for the defect in Δ*Moemc2* (Fig 5A).

To determine whether the phosphorylated MoRgs1 can rescue the invasive growth defect of the Δ*Moemc2* mutant, we performed injection assays on rice sheath and performed a microscopic observation. The results showed that the phosphorylated MoRgs1 partly rescues the IH growth of the Δ*Moemc2* mutant (Fig 5B). Moreover, we tested the virulence of *M*. *oryzae* by rice leaves spraying and rice sheath injection assays. Consistent with IH growth, MoEmc2 is required for the virulence of *M*. *oryzae* (Fig 5C and 5D). Growth, conidiation, biomass, penetration, and collapsed appressorium rate were all evaluated by activating the phosphorylation of MoRgs1 in the Δ*Moemc2* mutant, and the MoRgs1^5D^ partly suppresses the defect of the Δ*Moemc2* mutant (S3 Text). All of these results indicated that MoEmc2 is required for G-protein cAMP signaling to regulate appressorium formation and pathogenicity of *M*. *oryzae*.

### MoEmc2 is localized at the ER, late endosome, and PM

To examine the subcellular localization of MoEmc2 at the conidia and germ tube hooking stages during infection, we employed the Dye ER-Tracker to image ER, FM4-64 to label the PM and trafficking vesicles [56–58], and the late endosome (LE) marker protein GFP-MoRab7. Conidia and hooking germ tubes were observed and photographed by LSM. The co-localization was evaluated by the fluorescence intensity curve. All of the results suggested that, except for ER localization, MoEmc2 still locates at the late endosome and the inner plasma membrane at the conidia and germ tube hooking stages (S11 Fig).

To further determine whether MoEmc2 targets or migrates through the intracellular membrane, we treated MoEmc2-GFP transformants with ER-Golgi trafficking inhibitors brefeldin A (BFA) and 4-bromobenzaldehyde N-2, 6-dimethylphenyl (EGA), which is known as an inhibitor of early endosome (EE) to LE transport [28, 59–61]. After observing hundreds of conidia, the fluorescence of MoEmc2 was observed at the inner PM, indicating no MoEmc2 trafficking between the ER and the PM (S12A and S12B Fig). In addition, EGA treatment did not co-localize MoEmc2 with the EE marker MoRab5, following Pearson correlation coefficient analysis with ImageJ Coloc2 (S12C and S12D Fig). The results indicated both inhibitors have no effect on the subcellular localization of MoEmc2, suggesting that MoEmc2 is a membrane component of ER, LE, and inner PM organelles.

## Discussion

G-protein/cAMP signaling is required for appressorium formation and pathogenicity in the rice blast fungus *M*. *oryzae* [28, 62, 63]. The regulators of G-protein signaling, in particular MoRgs1, also plays an important role in these function [21]. As RGS proteins have a conserved function by modulating Gα activities through their intrinsic GAP functions, we here explored MoRgs1 regulatory mechanisms. We have demonstrated that the MoRgs1 GAP function requires MoCk2-dependent phosphorylation. The loss of phosphorylation resulted in an attenuation in the GAP function and dysregulated intracellular cAMP levels that are similar to those of the Δ*Morgs1* mutant. Sustainable phosphomimetic MoRgs1 could suppress the defect and restore intracellular cAMP levels to the extent of the wild type and complemented strains, even though it does not interact with the GDP-bound Gαs. This is consistent with that, following accelerated GTPase hydrolysis of Gαs, MoRgs1 dissociates from the GDP-bound Gαs. We have also found that MoEmc2, as an EMC subunit, modulates the subcellular localization of MoCk2 through a direct interaction, which also regulates MoRgs1 phosphorylation. In addition, we have provided evidence that the balance of MoRgs1 phosphorylation is equally important for appressorium development and pathogenicity of *M*. *oryzae*.

Phosphorylation is an important cellular regulatory mechanism that is widely involved in most cellular processes, including cell growth, development, division, signal transduction, and protein synthesis [64]. A previous study identified four phosphorylation sites S159, S399, S401, and S510 in MoRgs1 and suggested that hyper-phosphorylated MoRgs1 may be essential for pathogenicity [34]. We here identified additional phosphorylation sites by MoCk2 and that MoCka1 and MoCkb2 subunits are directly involved in this phosphorylation of MoRgs1. The newly identified five MoCk2-dependent serine phosphorylation sites S396, S399, S585, S696, and S700 are not in consensus with the minimum canonical sequence of Ser-Xaa-Xaa-acidic (S represent for serine, and X can be any amino acid) for the CK2 target site [65, 66]. It should be noted that this study examined only the direct subunits of MoCk2 in MoRgs1 phosphorylation.

As one of the core subunits of yeast EMC, Emc2 is usually recognized to promote the post-translational insertion of tail-anchored (TA) membrane protein with its first transmembrane domain (TMD) into the ER and biogenesis [44, 50, 53, 67]. Except for ER localization, MoEmc2 is also located at the late endosome and the inner PM at the conidia and germ tube hooking stages. This multicellular organelle localization indicates that MoEmc2 may have multiple roles, including that in directly regulating signal transduction. To further explore whether MoEmc2 directly anchors the organelle membrane components or shuffles protein trafficking between multicellular organelles, we employed the ER-Golgi trafficking inhibitor BFA and early to late endosome inhibitor EGA that disrupt protein trafficking. Both BFA and EGA treatment had no effects, suggesting MoEmc2 directly integrates with the internal membrane components without undergoes dynamic trafficking.

Previous studies also indicated that EMC promotes phospholipid transfer during lipid metabolism [49], which may satisfy the requirement during appressorium maturation and appressorial host penetration [17, 18]. Consistent with this, we found that MoEmc2 is critical for proper turgor pressure [17, 18, 68]. Intriguingly, we found a discrepancy in that intracellular cAMP levels in the Δ*Moemc2* strain were lower despite that MoEmc2 has a positive role in promoting MoRgs1 phosphorylation. We propose that the roles of MoEmc2 in regulating G-proteins signaling may be complexed, and additional studies are needed to dissect these roles.

**Fig 6:**
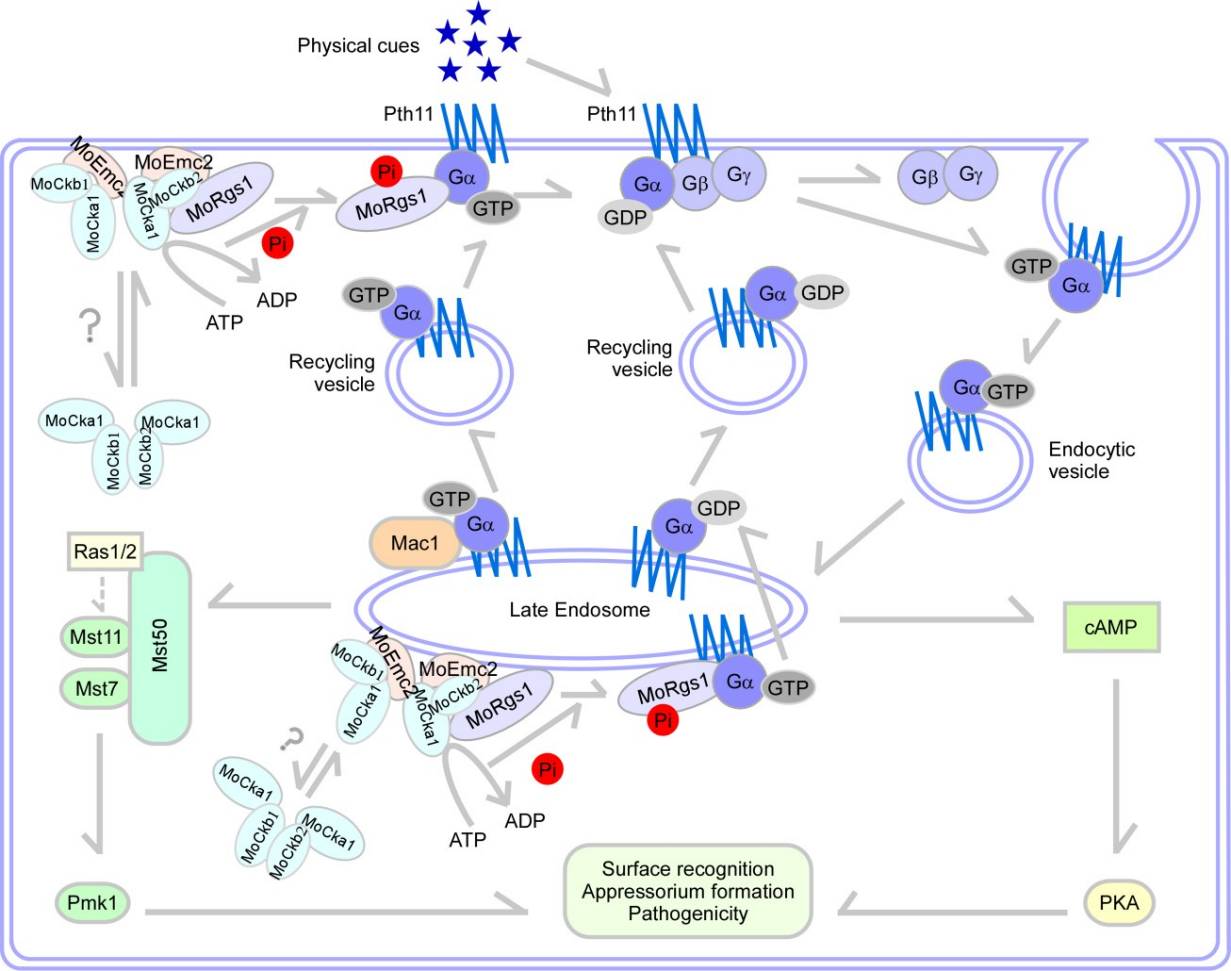
A proposed model of MoRgs1-MoEmc2 interaction in G-proteins signaling. In the process of recognizing hydrophobic cues at the surface of host plant rice, the ER membrane protein complex subunit MoEmc2 plays a critical role in modulating Gα function and cAMP signaling of *M*. *oryzae*. MoRgs1 is phosphorylated by MoCk2, then functions as a GTPase-activating protein (GAP) to negatively regulate G-protein signaling. At the same time, MoEmc2 regulates the subcellular localization of MoCk2 nearby the inner PM, which is critical for the MoRgs-MoCk2 interaction. The entire process is required for proper G-protein/cAMP signaling involved in appressorium formation and pathogenicity of *M*. *oryzae*.

In summary, the rice blast fungus *M*. *oryzae* senses the physical cues and activates G-proteins signaling during its interaction with the host. MoRgs1 plays an important function in modulating MoMagA-mediated cAMP signaling for appressorium formation and pathogenicity. In addition, MoRgs1 is subject to MoCk2-dependent protein phosphorylation, while this phosphorylation is also modulated by MoEmc2 (Fig 6). This multiple levels and complex regulatory circuitry likely ensure a proper balance between signal transduction and cellular homeostasis required for being a pathogen microorganism.

## Materials and methods

### Strains and culture conditions

The *M*. *oryzae* Guy11 was used as the wild-type strain for transformation, and all strains were cultured on complete medium (CM) plates at 28°C for 7 d [22]. For vegetative growth, small mycelium blocks were collected from 4-day-old colonies and transferred into fresh media, followed by incubation at dark 28°C. The radial growth was measured following incubation for 7 days. Mycelia were harvested from liquid CM medium and used for DNA, RNA tests, and protein extractions. For conidiation, mycelium blocks were maintained on SDC (100 g of straw, 40 g of corn powder, 15 g of agar in 1 L of distilled water) agar media at 28°C for 7 days in the dark followed by 3 days continuous illumination under fluorescent light.

### Phylogenetic tree analysis and yeast complementation assays

The proteins with high query coverage to MoEmc2 were acquired from the NCBI database website (https://www.ncbi.nlm.nih.gov/). Amino acid sequence alignment was made with CLUSTAL W analysis and the phylogenetic tree was built by MEGA 7.0. To test the homology of *MoEMC2* with *ScEMC2*, the full-length cDNA of *MoEMC2* was constructed with vector pYES2 (Invitrogen). Following PCR sequencing and screening on SD medium without uracil, the *MoEMC2*-pYES2 constructs were transformed into the yeast Δ*Scemc2* mutant (BY4741 loss of YJR088C). Yeast colonies were cultured on YPD medium (10 g Yeast Extract; 20 g Peptone 20 g D-Glucose) and adjusted to OD600 value 0.1. Followed by 10-fold serial dilutions were grown on SD-Met-Leu-His-Ura (galactose) plates at 30°C for 4 d and photographed.

### Targeted *MoEMC2* deletion and Δ*Moemc2* complementation

The *MoEMC2* gene deletion mutant was conducted by using the standard one-step gene replacement strategy. First, two approximate 1.0 kb of sequences flanking of *MoEMC2* were amplified with two primer pairs, the products of MoEMC2 were digested with restriction endonucleases (*Xho*I and *Eco*RI, *Bam*HI and *Spe*I) and ligated with hygromycin-resistance cassette (*HPH*) released from pCX62. The protoplasts of WT for targeted gene deletion were transformed with vectors with the hygromycin resistance marker gene cassette in which the marker gene was inserted into the middle of two flanking sequences of the *MoEMC2* gene. For screening hygromycin-resistant transformants, CM plates were supplemented with 250 μg/ml hygromycin B (Roche, USA). We generated the complementation construct pYF11-*MoEMC2*, the gene sequence containing the full-length of the *MoEMC2* gene gDNA sequence and 1.0 kb long native promoter with GFPEMC2F/GFPEMC2R. A yeast strain XK1-25 was co-transformed with the recombinant sequence and *Xho*I-digested pYF11 empty vectors. After 3–5 days of transformation and incubation, we screened the positive yeast transformants with polymerase chain reaction (PCR). Then we transformed the positive reconstructive plasmid into *E*. *coli* and with PCR test for acquiring positive clone of pYF11-*MoEMC2*. To generate the complemented strains, the pYF11-*MoEMC2* construct containing the bleomycin-resistant (BLM) gene was introduced into the Δ*Moemc2* mutant for *M*. *oryzae* transformants screening.

### Appressorium formation and virulence assays

We harvested conidia and filtered them through two layers of Miracloth (EMD Millipore Corporation, 475855-1R) with distilled water. For appressorium formation assays, droplets (30 μl) of conidial suspension were placed on plastic coverslips (Fisher Scientific, St Louis, MO, USA) or GelBond PAG film (GE Healthcare, 80112936) under humid conditions at 28°C [26]. All the samples were observed under Zeiss Axio Observer A1 inverted microscope (40 ×).

For virulence assays, 0.2% (w:v) gelatin was added to conidia suspension (5 × 10^4^ spores/ml) to promote adhesion. Two weeks old seedlings of rice (*Oryza sativa* cv. CO39) were used for rice spraying assays. We inoculated 5 ml of conidial suspension each treatment onto rice seedlings with a sprayer. We kept inoculated seedlings in a growth chamber at 28°C with 90% humidity, and the first 24 h was in the dark, followed by a 12 h -12 h light-dark cycle. The degree of disease was judged at 7 days post-inoculation. The degree of disease lesions was analyzed by ImageJ.

For the sheath injection assays *in vivo*, conidia suspensions (2 × 10^5^ spores/ml) were injected into 3-week-old rice sheath with syringes and incubated in the same conditions as rice spraying assays. After 5 days post-inoculation, tissues that emerge from the sheath were collected and photographed. For the penetration assays *in vitro*, the conidia suspension (2 × 10^5^ spores/ml) was injected into 3-week-old rice sheath with syringes and harvested after 36 h post-incubation at dark 28°C. Epidermal cells in the leaf sheath were removed for microscopic observation. Each experiment was repeated with three biological repetitions and three replicates under the same experimental conditions (e.g., temperature, humidity, illumination, and phase of host plants).

### Protein extraction and western blot analysis

For total protein extraction, strains were cultured in liquid CM media with shaking for 36 h. Mycelia were collected and ground into fine powder, then resuspended in 1ml triton lysis buffer (10 mM Tris-HCl, pH 7.5, 150 mM NaCl, 0.5 mM EDTA (Solarbio, E8030), 0.5% NP-40 [Sigma-Aldrich, IGEPAL CA-630, I3021]) with 2 mM PMSF and proteinase inhibitor cocktail (Sigma-Aldrich, cOmplete, 11836170001). The lysates were collected into 2.0 ml EP tubes and placed onto the ice for 30 min and shaken every 10 min. Protein was extracted by centrifugation at 15,000 rpm for 10 min at 4°C and collecting the supernatant lysates as total proteins. For GFP-tagged protein detection, samples were analyzed by 12% SDS-PAGE gel with running immunoblotting and combining anti-GFP antibody (mouse, 1:5000, Abmart, 293967) and the anti-mouse secondary antibody (1:10,000, LI-COR, IRDye, C70301-02), detected by ODYSSEY infrared imaging system (software Version 2.1). For S-tagged protein detection, samples were analyzed with anti-S antibody (rabbit, 1:5000, Abcam, 19369) and the anti-rabbit secondary antibody (1:10,000, LI-COR, IRDye, C90723-19). For RFP-tagged protein detection, samples were analyzed with anti-RFP antibody (mouse, 1:5000, Chromotek, 6g6-100) and the anti-mouse secondary antibody (1:10,000, LI-COR, IRDye, C70301-02). For detecting phosphorylated Pmk1 level, we used anti-phospho-p44/42 MAPK (Erk1/2) antibody (Cell Signaling Technology, 4370) with anti-p44/42 MAP kinase antibody (Cell Signaling Technology, 4695) as control.

### Construction of the MoMagA-RFP plasmid vector

Tagging at the C terminal or the N terminal of MoMagA is known to jeopardize the Gα function, but a previous report indicated that introducing a tag peptides coding sequence in-frame into the αB-αC loop of MoMagA would effectively address this issue [30]. We thereby introduced an RFP fusion into MoMagA between amino acids 113–120.

### Yeast two-hybrid assays

Bait constructs were conducted by cloning full-length cDNAs of target genes into pGADT7 and pGBKT7 (LMAI Biotechnology, LM1010). Prey constructs and bait constructs were confirmed by DNA sequencing and co-transformed into the yeast strain AH109 (LMAI Biotechnology, LM1010) following the recommended protocol (BD Biosciences Clontech). Transformants screened by synthetic dextrose medium minus leucine, tryptophan, adenine, and histidine (SD-Leu-Trp-Ade-His) were selected. Yeast strains for positive and negative controls were provided by the BD library construction and screening kit.

### Co-immunoprecipitation (co-IP) assays

To confirm the interactions between MoRgs1 with all three MoCk2 holoenzyme subunits MoCka1, MoCkb1, and MoCkb2 *in vivo*, full-length gDNA with 1500 bp native promoter region of *MoCka1*, *MoCKB1*, and *MoCKB2* were cloned into pXY203, a vector with a S tag [69]. Full-length gDNA of *MoRGS1* was constructed on pYF11 labelled by the GFP tag [69]. All constructs were introduced into the protoplasts of WT strains Guy11 by co-transformation. Total proteins were extracted from transformants co-expressing MoRgs1-GFP with MoCka1-S, MoCkb1-S, and MoCkb2-S, then incubated with anti-GFP affinity beads (Smart lifesciences, SA070001). Proteins bound to the beads were eluted after a series of washing steps by 1 × PBS (diluted from 10 × PBS [Beyotime Biotechnology, ST476]). Elution buffer (200 mM glycine, pH 2.5) and neutralization buffer (1 M Tris base, pH 10.4) was used for the elution process. A similar method was used to test interactions among MoRgs1-GFP with MoEmc2-S, MoEmc2-GFP with all three MoCk2 holoenzyme subunits MoCka1-S, MoCkb1-S, and MoCkb2-S, and MoMagA-RFP with MoRgs1-GFP, MoRgs1^5A^-GFP, and MoRgs1^5D^-GFP, respectively. The interactions among MoMagA with MoRgs1, MoRgs1^5A^ and MoRgs1^5D^ were purified by anti-RFP affinity beads (Chromotek, rta-20).

### Bimolecular fluorescence complementation (BiFC) assays

The protein MoRgs1, MoCka1, MoCkb1, MoCkb2, and MoEmc2 fusion constructs were generated by cloning full-length gDNA with 1500 bp native promoter region into pHZ65 and pHZ68 (BiFC vectors were graciously provided by Dr. J. R. Xu of Purdue University, USA), respectively [70]. Constructs pairs of MoRgs1-YFP^N^ and all three MoCk2 holoenzyme subunits MoCka1-YFP^C^, MoCkb1-YFP^C^ and MoCkb2-YFP^C^ were co-introduced into the protoplasts of WT strain, respectively. Transformants screened by two antibiotic hygromycin (Solarbio Life Sciences, H8080) and zeocin (Thermo Fisher Scientific, R25001) were isolated and confirmed by PCR analyses. Similar methods were used to detect interactions of MoEmc2-YFP^N^ with MoRgs1-YFP^C^ and all three MoCk2 holoenzyme subunits MoCka1-YFP^C^, MoCkb1-YFP^C^, and MoCkb2-YFP^C^, respectively.

### GST-pull down

GST, GST-MoCka1, GST-MoCkb1, GST-MoCkb2 and His-MoRgs1 were expressed in *Escherichia coli* BL21-CodonPlus (DE3) cells (Sigma, CMC0014). Cells were lysed in lysis buffer (50 mM Tris, pH 8.0, 50 mM NaCl, 1 mM PMSF [Beyotime Biotechnology, ST506-2]) with a sonicator (Branson). Samples were centrifuged (13,000 g, 10 min) and the supernatants were transferred to a new 1.5 ml tube and stored at 70°C. The GST, GST-MoCka1, GST-MoCkb1 and GST-MoCkb2 supernatants were then mixed with 30 ml glutathione sepharose beads (GE Healthcare, 10265165) and incubated at 4°C for 2 h. The recombinant GST, GST-MoCka1, GST-MoCkb1, and GST-MoCkb2-bound to glutathione sepharose beads were incubated with *E*. *coli* cell lysate containing His-MoRgs1 at 4°C for another 4 h. Finally, the beads were washed with buffer (50 mM Tris, pH 8.0, 50 mM NaCl, 1 mM PMSF, 1% Triton X-100) five times and eluted from the beads. Eluted proteins were then analyzed by immunoblot (IB) with monoclonal anti-His and monoclonal anti-GST antibodies, respectively.

### Assays under confocal laser scanning microscope observation

All strains transformed with fluorescent labeling were observed under the confocal laser scanning microscope (Zeiss LSM710, 63 × oil). The filtered channels set: GFP (excitation spectra: 488 nm, emission spectra: 510 nm), RFP (excitation spectra: 555 nm, emission spectra: 584 nm), YFP (excitation spectra: 514 nm, emission spectra: 528 nm). Exposure time: 800 ms. Insets highlight areas analyzed by line-scan. Bar = 10 μm. ImageJ software was applied to calculate the Pearson correlation coefficient for analyzing co-localization of GFP fusion protein with RFP fusion protein if needed. One area of interest was photographed with GFP and RFP channels, respectively. Photographs were opened using ImageJ. Image type was set to 8 bits, respectively. The ‘Colocalization Coloc2’ in the ‘Analysis’ section was applied to all images.

### Intracellular cAMP level measurement by HPLC

*M*. *oryzae* strains or transformants were harvested in liquid medium CM for 48 h. Samples were lyophilized for 24 h and grounded into fine powders in liquid nitrogen. Intracellular cAMP was extracted following previously established procedures [21]. The intracellular cAMP levels were quantified by HPLC (High-Performance Liquid Chromatography). The relevant experimental procedures were assessed, as described previously [71].

### *In vivo* phosphorylation assays using Phos-tag gel electrophoresis

The MoRgs1-GFP fusion construct was introduced into WT, Δ*Mockb1*, Δ*Mockb2*, and Δ*Moemc2* strains. The total protein extracted from mycelia was resolved on 8% SDS-PAGE prepared with 50 μM acrylamide-dependent Phos-tag ligand and 100 μM MnCl_2_ as described [72, 73]. Gel electrophoresis was performed with a constant voltage of 80 V for 6 h. Before transferring, gels were equilibrated in transfer buffer with 5 mM EDTA for 20 min two times and followed by transfer buffer without EDTA for another 20 min. Protein transferred from the Mn^2+^-phos-tag acrylamide (NARD institute Limited company, 18D-01) gel to the PVDF membrane (EMD Millipore Corp., ISEQ00010) was performed for 40 h (depend on different proteins) at 80 V at 4°C, then it was analyzed by immunoblotting using the anti-GFP antibody.

### *In vitro* phosphorylation analysis

GST-MoCka1, GST-MoCkb1, GST-MoCkb2, His-MoRgs1, and His-MoRgs1^5A^ were expressed in *E*. *coli* DE3 cells and purified. A rapid and cost-effective fluorescence detection in tube (FDIT) method was used to analyze *in vitro* protein phosphorylation [74]. The Pro-Q Diamond Phosphorylation Gel Stain (Thermo Fisher Scientific, P33301) is used for phosphor-protein gel-staining. For protein kinase reactions, 2 μg MoRgs1 (MoRgs1^5A^) was mixed with MoCka1, MoCkb1, and MoCkb2 in a kinase reaction buffer (100 mM PBS [Beyotime Biotechnology, ST476], pH 7.5, 10 mM MgCl_2_, 1 mM ascorbic acid [Sigma-Aldrich, A5960]), with the appearance of 50 μM ATP (Sigma-Aldrich, FLAAS) at room temperature (RT) for 60 min, 10 folds of cold acetone was added to stop the reaction. For protein in tube staining, samples were homogenized and suspended in Mili-Q water at the concentration of 0.2 μg/μl. The pellet was rinsed with 0.5 ml cold acetone and centrifuge to remove the supernatant twice. The pellet was air-dried and dissolved in 200 μl of Mili-Q water and moved to a black 96 well plate (Corning, 3925). Fluorescence signal at 590 nm (excited at 530 nm) was measured in a Cytation3 microplate reader (Biotek, Winooski, VT, USA) [73].

### LC-MS-MS analysis

To identify phosphorylation sites of the substrate, proteins were extracted and separated on 10% SDS-PAGE. In-gel digestion (or elution digestion), the stained protein bands were in-gel-digested after overnight incubation in a destaining solution, followed by drying in an acetonitrile solution and equilibration in 20 mM ammonium bicarbonate. The liquid was removed, and pellets were washed by acetonitrile and dehydrated in a speed vac until protein bands are completely opaque. Proteins were reduced with 10 mM DTT at 56°C for 1 h and alkylated immediately using 55 mM iodoacetamide (IAM) in the dark at room temperature for 1 h. Liquid removal, acetonitrile wash, and dehydration were again performed. Trypsin solution (1 μg/μl in 20 mM ammonium carbonate, pH 8.9) was added, and then a buffer was added until the protein bands were restored in size (rehydrated). The digestions were carried out overnight at 37°C. The resulting peptides were recovered by two extractions of 20 min each, with 100 μL of a solution of 60% acetonitrile acid. When the highly hydrophobic peptides were expected, the third extraction with 60% acetonitrile plus 0.1% formic acid was performed. The extracts were combined and concentrated to ~ 20 μL in a speed vac.

Each fraction was resuspended in buffer A (2% ACN, 0.1% FA) and centrifuged at 20,000 g for 15 min, 10 μl supernatant was loaded onto an Acclaim PePmap C18-reversed-phase column(75 μm × 2 cm, 3 μm, 100 Ǻ thermo scientific) and separated with reversed-phase C18 column (75 μm × 10 cm, 5 μm, 300 Ǻ, Agela Technologies) mounted onto a Dionex ultimate 3000 nano LC system. Peptides were eluted using the following gradient scheme: 0 ~ 6 min 5%-8% Buffer B; 6 ~ 40 min 8–30% Buffer B; 40 ~ 45 min 30–60% Buffer B; 45 ~ 48 min 60–80% Buffer B, 48–56 min 80% Buffer B; 56–58 min 80–5% Buffer B (decreasing to 5%); 58–65min 5% Buffer B. at a flow rate of 400 nL·min^-1^ combined with a Q Exactive mass spectrometer (Thermo Fisher Scientific, MA, USA). The eluates were directly entered Q-Exactive MS (Thermo Fisher Scientific, Waltham, MA, USA), setting in a positive ion mode and data-dependent manner with full MS scan from 350–2000 m/z, full scan resolution at 70,000, MS/MS scan resolution at 17,500. MS/MS scan with minimum signal threshold 1E+5, isolation width at 2 Da. To evaluate the performance of this mass spectrometry on the labeled samples, two MS/MS acquisition modes, higher collision energy dissociation (HCD) were employed. And to optimize the MS/MS acquisition efficiency of HCD, normalized collision energy (NCE) was systemically examined 28, stepped 20%.

Peptide identification and quantification were carried out on the Mascot software Revision 2.3.01 using the TAIR database search algorithm and the integrated false discovery rate (FDR) analysis function. For mass spectrometry analyzing the phosphorylation sites, we thank the Beijing Protein Innovation Co., Ltd., for technological assistance.

### GTPase activity assays

The fusion constructs His-MoRgs1, His-MoRgs1^5A^, His-MoRgs1^5D^, and His-MoMagA were expressed in *E*. *coli* DE3 cells and purified. Then we conducted GTPase activity assays with the ATPase/GTPase Activity Assay kit (MAK113; Sigma-Aldrich; Merck KGaA, Darmstadt, Germany) instructions. First, the phosphate standards were set as indicated in the kit instructions to plot a standard curve. Second, a series of dilutions of purified proteins were performed in assays buffer. The sample reactions and the control well were set up according to the scheme. The reaction was incubated for the desired period of time (10 s, 30 s, 1 min, 2 min, 5 min, and 10 min) at room temperature. Reagent (200 ml) was added to each well, and incubation was carried out for an additional 30 min at room temperature to terminate the enzyme reaction and generate the colorimetric product separately. Absorbance at 600–660 nm [maximum absorbance at 620 nm (A620)] was read. We calculated the change in absorbance values (DA620) for the samples by subtracting the A620 of the control well (A620) control from the A620 of the sample well (A620) sample. The concentration (mM) of free phosphate [Pi] was computed in the sample from the standard curve. The formula was: Enzyme activity (units/l) = [Pi] (mM) × 40 ml ÷ [10 μl × reaction time (min)]. One unit is the amount of enzyme that catalyzes the production of 1 mmol of free phosphate per minute under the assay conditions. The relevant experimental procedures were assessed, as described previously [75].

### Statistical analysis

Each experiment was performed with three replicates and represented as the mean ± standard deviation (SD). The significant differences between treatments were statistically determined by one-way analysis of variance (ANOVA) comparison and followed by Duncan’s new multiple range tests if the ANOVA analysis is significant at *P* < 0.01 as described previously [76].

### Accession numbers

Genes reported in this article can be found in the GenBank database under the following accession numbers: MoRgs1 (MGG_14517), MoEmc2 (MGG_07480), MoCka1 (MGG_03696), MoCkb1 (MGG_00446), MoCkb2 (MGG_05651), MoMagA (MGG_01818), MoMagB (MGG_00365), MoMagC (MGG_04204), MoMgb1 (MGG_05201), MoMgg1 (MGG_10193), MoRab5 (MGG_06241), MoRab7 (MGG_08144), Mst7 (MGG_00800), MoMac1 (MGG_09898).

## Supporting information

S1 PRIMERS Checklist Primers used in this study. (DOCX)

S1 Fig MoRgs1 is phosphorylated during the developmental and invasive stages of *M*. *oryzae*. MoRgs1-GFP proteins were extracted from transformants at mycelia, conidia, and appressoria stages then treated with phosphatase and phosphatase inhibitors. Mn^2+^-Phos-tag SDS-PAGE and normal SDS-PAGE were used to conduct Western blot analysis with the anti-GFP antibody. The extent of MoRgs1 phosphorylation was estimated by the mobility shift assay.
(TIF)

S2 Fig MoCk2 interacts with MoRgs1. (A) The interaction among all three subunits of MoCk2 holoenzyme (GST-MoCka1, GST-MoCkb1, and GST-MoCkb2) with His-MoRgs1 were conducted by GST pull-down assays. GST-MoCka1, GST-MoCkb1, GST-MoCkb2, His-MoRgs1, and GST were expressed and purified by affinity chromatography. Bound proteins were separated by SDS-PAGE in duplicate and analyzed by Western blot with the anti-His (Mouse; M20001; Abmart) and anti-GST antibodies (Mouse; M20007; Abmart). (B) The interaction among all three subunits of MoCk2 holoenzyme (MoCka1-YFP^C^, MoCkb1-YFP^C^, and MoCkb2-YFP^C^) with MoRgs1-YFP^N^ were conducted by BiFC. Empty YFP^C^ and empty YFP^N^ constructs were used as a negative control. The co-transformants were observed at the germ tube hooking stage (3 h) with laser scanning microscopy (Zeiss LSM710 laser scanning microscope, 63 × oil, Bar = 10 μm). The mean and standard deviation of fluorescence intensity were denoted over 50 germinated conidia that were randomly chosen.
(TIF)

S3 Fig MoCkb1 is dispensable for MoRgs1 phosphorylation. (A) Phosphorylation analysis of MoRgs1 *in vivo*. Total proteins treated with phosphatase and phosphatase inhibitors were detected by the GFP antibody. Bands were shifted by Mn^2+^-Phos-tag SDS-PAGE and normal SDS-PAGE, respectively. (B) Phosphorylation analysis *in vitro* by the fluorescence detection in tube (FDIT) method. Purified proteins of GST-MoCka1, GST-MoCkb1, GST-MoCkb2, and His-MoRgs1 were constructed for the protein kinase reaction in the presence of ATP. Fluorescence was measured in a microplate reader (**P < 0.01, n = 3).
(TIF)

S4 Fig Identification of MoRgs1 phosphorylation sites by LC-MS-MS (Q-E). (A-E) Identified differentiated phosphorylation sites of MoRgs1 between the WT and Δ*Mockb2* strains by LC-MS-MS (Q-E). The five phosphorylation sites (S396, S399, S585, S696, and S700) were identified in the wild type strain. Phosphorylation site sand sequences are annotated in the upper panel. (F) A model of five serine sites located at the MoRgs1 domains. (G) Covered peptides and phosphorylated sites in the wild type, Δ*Mockb2*, and Δ*Moemc2* strains. Colorful letters represent the amino acid sequences covered by mass spectrometry. Red letters represent phosphorylation sites newly identified.
(TIF)

S5 Fig Unphosphorylated MoRgs1 interacts with the GDP-bound MoMagA but not phosphomimetic MoRgs1. Co-IP analysis for the interaction between MoMagA and MoRgs1, MoRgs1^5A^, and MoRgs1^5D^, respectively. Total proteins were extracted and incubated with the anti-GFP agarose and then eluted for Western blot analysis using anti-RFP or anti-GFP antibodies.
(TIF)

S6 Fig Phylogenetic analysis and yeast complement with MoEmc2. (A) The amino acid sequences of diverse Emc2 proteins from corresponding organisms were aligned using the CLUSTAL_W. The neighbor-joining tree was constructed by MEGA 7.0 with 1000 bootstrap replicates. GenBank accession numbers and the corresponding species names are as listed: XP_003711387.1 (*Magnaporthe oryzae* MoEmc2), NP_012621.1 (*Saccharomyces cerevisiae* ScEmc2), KUI71153.1 (*Valsa mali* TPR repeat protein), PTD09165.1 (*Fusarium culmorum* TPR repeat protein), KZL69988.1 (*Colletotrichum tofieldiae* TPR repeat protein), XP_009648592.1 (*Verticillium dahliae* TPR repeat protein), OQE20945.1 (*Penicillium flavigenum* TPR repeat protein), TBU37051.1 (*Dichomitus squalens* TPR-like protein), NP_850995.1 (*Arabidopsis thaliana* AtPpts), and NP_055488.1 (*Homo sapiens* HsEmc2). (B) *MoEMC2* suppressed the heat sensitivity of the yeast Δ*emc2* strain. 10-fold serial dilutions of BY4741, Δ*Scemc2*, and Δ*Scemc2* transformed with pYES2-*MoEMC2* constructs were grown on SD-Met-Leu-His-Ura (galactose) plates at 30°C and 37°C for 4 days and then photographed.
(TIF)

S7 Fig The N-terminus of MoEmc2 interacts with the N-terminus of MoRgs1. (A) Structure and domain prediction of MoEmc2 using SMART (http://smart.embl-heidelberg.de/). The positions of the domains within the proteins were indicated by amino acid numbers. The full length of *MoEMC2* was divided into NTD, TPR, and CTD domains before being ligated in pGBKT7. (B) MoRgs1 has two DEP domains at the N-terminus and one RGS domain at the C-terminus [22, 31]. Similar methods were used to conduct the following MoRgs1 vectors in pGADT7: AD-MoRgs1, AD-N-Rgs1, and AD-C-Rgs1. (C) The full length and regions of MoRgs1 and MoEmc2 were assayed by Y2H. The yeast co-transformants expressing the bait and prey constructs were isolated on the SD-Leu-Trp plate for 3 d and screened by SD-Ade-His-Leu-Trp plates for 5 d.
(TIF)

S8 Fig *MoEMC2* mutant transformants were confirmed by Southern blot analysis. (A) A model of the *MoEMC2* gene deletion by homologous recombination in *M*. *oryzae*. (B) Gene-specific probe (probe 1) and hygromycin phosphotransferase (*HPH*) probe (probe2) were used in Southern hybridization. Thick square frames indicate the sites of *MoEMC2* and *HPH* genes. Thin lines below the square frames indicate sequence-specific gene probes.
(TIF)

S9 Fig MoEmc2 regulates the subcellular localization of MoCkb1 and the interaction between MoCkb1 and MoRgs1. (A and B) Fluorescence GFP labeled MoCkb1-GFP, and MoRgs1-GFP fusion constructs were introduced into the WT and Δ*Moemc2* strains at the germ tube hooking stage (3 hpi). Insets highlight areas analyzed by line-scan. Bar = 10 μm. Percentage of a pattern showed in image was calculated by observation for 50 germinated conidia that were randomly chosen, and observation was conducted for 3 times. (C) Co-IP assays for the interaction between MoRgs1-GFP with MoCkb1-S in the WT and Δ*Moemc2* strains. Total proteins were extracted and eluted from the anti-GFP agarose beads before being analyzed by immunoblotting with corresponding antibodies. T: Total protein E: Elution.
(TIF)

S10 Fig MoEmc2 is required for appressorium formation and pathogenicity in *M*. *oryzae*. (A and B) Rice spraying assays and lesion area statistics. Conidial suspensions (5 × 10^4^ spores/ml) were sprayed onto 2 week-old rice seedlings (CO-39). Diseased rice leaves were photographed and percentages of per 5 cm length leaf lesion area were analyzed by ImageJ after 7 days of inoculation. Values are means of three replications and SD (**P < 0.01, n = 10). (C and D) Rice sheath injecting assays *in vivo* and lesion area statistics. Conidial suspensions (2 × 10^5^ spores/ml) were sprayed onto 4 week-old rice seedlings (CO-39). Diseased rice leaves were photographed and percentages per 5 cm length leaf lesion area were analyzed by ImageJ after 5 days of inoculation. Values are means of three replications and SD (**P < 0.01, n = 10). White triangles point out the injection sites. (E and F) Rice sheath injecting assays *in vitro* and classification statistics. Invasive hyphae (IH, n = 100) in rice cells were observed at 36 hpi and 4 types of were quantified and statistically analyzed. Error bars represent SD from three independent replicates. (G and H) Appressorium formation assays and statistics analysis. Conidia of the WT, Δ*Moemc2* and complemented Δ*Moemc2* (Δ*Moemc2/MoEMC2*) strains were dropped on hydrophobic surfaces and the dynamics of appressorium formation were photographed at various times (**P < 0.01, n = 100). Bar = 10 μm. (I) Appressorium formation was assayed on hydrophobic (the upper panel) and hydrophilic (the upper panel) surfaces for 24 hpi. Percentages of Mean and SD were shown at the lower panel. (J) Intracellular cAMP levels in the mycelia of the indicated strains cultured for 2 d in CM were quantified by HPLC (**P < 0.01, n = 3). (K) Morphological characteristics of the WT and Δ*Moemc2* strains. Percentages of Mean and SD were depicted at the lower panel (**P < 0.01, n = 100). Bar = 10 μm.
(TIF)

S11 Fig MoEmc2 is located at the endoplasmic reticulum, late endosome, and inner plasma membrane. (A and D) MoEmc2-GFP transformants were stained by endoplasmic reticulum dye ER-Tracker at conidia and germ tube hook stages. (B and E) MoEmc2-GFP transformants were stained by FM4-64 at the conidia and germ tube hooking stages (3 h). (C and F) Late endosome marker GFP-MoRab7 was co-transformed with MoEmc2-RFP in the WT strain and observation was made at the conidia and germ tube hooking stages. All assays were observed 100 samples with three replicates and insets highlight areas analyzed by line-scan. Bar = 10 μm.
(TIF)

S12 Fig BFA and EGA inhibitors fail to alter MoEmc2 localization. (A and B) ER-Golgi trafficking inhibitor BFA was used in conidium assays with DMSO solvent as control. Insets highlight areas analyzed by line-scan. Bars = 10 μm. (C and D) Early to late endosome inhibitor EGA assay was conducted in GFP-MoRab5 and MoEmc2-RFP co-transformants (3 h, germ tube hooking stage). Percentages of the pattern (shown in the images) were calculated by the observation of 100 randomly chosen germinated conidia, and the observation was conducted 3 times. The extent of fluorescence overlap was estimated with the Pearson correlation coefficient calculated by ImageJ coloc2. Mean and standard deviation were indicated in the right column (n = 30). Bars = 10 μm.
(TIF)

S1 Text Identification of MoRgs1 binding proteins. The bait construct AD-MoRgs1 was used to screen a yeast two-hybrid cDNA library constructed with an RNA pool from various stages, including conidia and infectious hyphae (0, 2, 4, 8, 12 and 24 h).
(DOCX)

S2 Text Phenotype analysis of the wild type, Δ*Morgs1* mutant, site-directed mutagenesis mutants, complement strains. a. Colony diameter of the indicated strains on CM and SDC media after 7 days incubation at 28°C. b. Dry weight of hyphal at 2 days after incubation in liquid complete medium at room temperature by shaken at 160 rpm. c. Quantification of the conidial production of the indicated strains formed on SDC cultures in the dark for 7 d, followed by incubation under constant illumination for 3 d at room temperature. d. Percentage of appressoria penetrating rice sheath epidermal cells at 24 h post-inoculation. All different capital letters in column show significant difference (Duncan’s new multiple range test,*P* < 0.01). All experiments were conducted with three biological repetitions and three replicates, mean and standard deviations were calculated. 5A: S396A S399A S585A S696A S700A 5D: S396D S399D S585D S696D S700D.
(DOCX)

S3 Text Phenotype analysis of the wild type (WT), Δ*Moemc2* mutant, Δ*Moemc2/MoRGS1*^5D^ and complement (Δ*Moemc2/MoEMC2*) strains. a. Colony diameter of the indicated strains on CM, OM, MM and SDC agar plates after 7 dark days incubation at 28°C. b. Dry weight of hyphae 2 days after incubation in liquid complete medium at 28°C with 160 rpm. c. Statistics of conidial production of the indicated strains growing 7 dark days on SDC agar plates followed by constant illumination (wavelength of 365 nm) for 3 d at room temperature. d. Percentage of appressorium formation on hydrophobic surface with 24 h post-inoculation. e. Percentage of collapsed appressorium treated with 1 M, 2 M, 3 M and 4 M glycerol solution for 5 min after appressorium formation with 24 hpi. All different capital letters in column show significant difference (Duncan’s new multiple range test, *P* < 0.01). All experiments were conducted with three biological repetitions and three replicates, mean and standard deviations were calculated.
(DOCX)
