# Supplementary material for: The rice blast fungus MoRgs1 functioning in cAMP signaling and pathogenicity is regulated by casein kinase MoCk2 phosphorylation and modulated by membrane protein MoEmc2
Source: PLoS Pathog. 2021 Jun 16;17(6):e1009657. doi: 10.1371/journal.ppat.1009657 (PMC8208561; doi:10.1371/journal.ppat.1009657)
Supplement: S1 PRIMERS Checklist — (DOCX) [file ppat.1009657.s001.docx]

S1 PRIMERS Checklist. Primers used in this study.

| Primer name | Sequence (5’-3’) | Remark |
| --- | --- | --- |
| ADRGS1F | CGCCATATGATGGACGACACCTCCCGCCCG | Construction of *pGADT7*-*MoRGS1* |
| ADRGS1R | CCGGAATTCTCATAACCGTTGCGAGCGGCT | Construction of *pGADT7*-*MoRGS1* |
| HisRGS1F | CCGGAATTCATGGACGACACCTCCCGCCCGGACC | Construction of *pET32*-*a*-*MoRGS1* |
| HisRGS1R | CCCAAGCTTTAACCGTTGCGAGCGGCTTTGGCTG | Construction of *pET32*-*a*-*MoRGS1* |
| CYFPRGS1F | CGACTCACTATAGGGCGAATTGGGTACTCAAATTGGCCTAAAAATGTATTCCTATTAAG | Construction of *MoRGS1*-*CYFP* |
| CYFPRGS1F | GTTCGGGATCTTGCAGGCCGGGCGTAACCGTTGCGAGCGGCTTTGGCTG | Construction of *MoRGS1*-*CYFP* |
| NYFPRGS1F | ACTCACTATAGGGCGAATTGGGTACTCAAATTGGCCTAAAAATGTATTCCTATTAAGA | Construction of *MoRGS1*-*NYFP* |
| NYFPRGS1R | GCTCACCATCGTGGCGATGGAGCGTAACCGTTGCGAGCGGCTTTGG | Construction of *MoRGS1*-*NYFP* |
| 5ARGS1F1 | TCACTATAGGGCGAATTGGGTACTCAAATTGGTTAGTCTAGAGTATAACTCATTGGTAC | Construction of *MoRGS1*^5A^ -*GFP* |
| 5ARGS1R1 | GCGGTAGTCACTTAATGAGTCCGCGTCGGCAGCGCTGACGCT | Construction of *MoRGS1*^5A^ -*GFP* |
| 5ARGS1F2 | AGCGTCAGCGCTGCCGACGCGGACTCATTAAGTGACTACCGC | Construction of *MoRGS1*^5A^ -*GFP* |
| 5ARGS1R2 | AGTTCGCCGGCGCGCTTTGGGCCAGCTGTG | Construction of *MoRGS1*^5A^ -*GFP* |
| 5ARGS1F3 | CACAGCTGGCCCAAAGCGCGCCGGCGAACT | Construction of *MoRGS1*^5A^ -*GFP* |
| 5ARGS1R3 | TACCCGCGGTGCTGACTCTAGAGCCACTGAGTCAAAGT | Construction of *MoRGS1*^5A^ -*GFP* |
| 5ARGS1F4 | ACTTTGACTCAGTGGCTCTAGAGTCAGCACCGCGGGTA | Construction of *MoRGS1*^5A^ -*GFP* |
| 5ARGS1R4 | GCGCAGAGGAGCGTGAATGTTGAGTGGAATGATTTACTTGTACAGCTCGTCCATGCCG | Construction of *MoRGS1*^5A^ -*GFP* |
| 5DRGS1F1 | TCACTATAGGGCGAATTGGGTACTCAAATTGGTTAGTCTAGAGTATAACTCATTGGTAC | Construction of *MoRGS1*^5D^-*GFP* |
| 5DRGS1R1 | GCGGTAGTCACTTAATGAGTCATCGTCGGCATCGCTGACGCT | Construction of *MoRGS1*^5D^-*GFP* |
| 5DRGS1F2 | AGCGTCAGCGATGCCGACGATGACTCATTAAGTGACTACCGC | Construction of *MoRGS1*^5D^-*GFP* |
| 5DRGS1R2 | AGTTCGCCGGATCGCTTTGGGCCAGCTGTG | Construction of *MoRGS1*^5D^-*GFP* |
| 5DRGS1F3 | CACAGCTGGCCCAAAGCGATCCGGCGAACT | Construction of *MoRGS1*^5D^-*GFP* |
| 5DRGS1R3 | TACCCGCGGATCTGACTCTAGATCCACTGAGTCAAAGT | Construction of *MoRGS1*^5D^-*GFP* |
| 5DRGS1F4 | ACTTTGACTCAGTGGATCTAGAGTCAGATCCGCGGGTA | Construction of *MoRGS1*^5D^-*GFP* |
| 5DRGS1R4 | GCGCAGAGGAGCGTGAATGTTGAGTGGAATGATTTACTTGTACAGCTCGTCCATGCCG | Construction of *MoRGS1*^5D^-*GFP* |
| 5AHisRGS1F1 | CCGGAATTCATGGACGACACCTCCCGCCCGGACC | Construction of *pET32*-*a*-*MoRGS1*^5A^ |
| 5AHisRGS1R1 | GCGGTAGTCACTTAATGAGTCCGCGTCGGCAGCGCTGACGCT | Construction of *pET32-a-MoRGS1*^5A^ |
| 5AHisRGS1F2 | AGCGTCAGCGCTGCCGACGCGGACTCATTAAGTGACTACCGC | Construction of *pET32*-*a*-*MoRGS1*^5A^ |
| 5AHisRGS1R2 | AGTTCGCCGGCGCGCTTTGGGCCAGCTGTG | Construction of *pET32*-*a*-*MoRGS1*^5A^ |
| 5AHisRGS1F3 | CACAGCTGGCCCAAAGCGCGCCGGCGAACT | Construction of *pET32*-*a*-*MoRGS1*^5A^ |
| 5AHisRGS1R3 | CCCAAGCTTTAACCGTTGCGAGCGGCTTTGGCTGCGTTCGGGTACCCGCGGTGCTGACTCTAGAGC | Construction of *pET32*-*a*-*MoRGS1*^5A^ |
| KOEMC2F1 | CCGCTCGAGGGGAGTGGCTCAGACGTGGCAGCAG | Amplifying MoEMC2 5' flank sequence for gene knock out |
| KOEMC2R1 | CCGGAATTCCTTGTCGGTCGGTCGGGCTTGCACA | Amplifying MoEMC2 5' flank sequence for gene knock out |
| KOEMC2F2 | CGCGGATCCGATAACCTACAGTATGCTCAGTCCA | Amplifying MoEND3 3' flank sequence for gene knock out |
| KOEMC2R2 | CGGACTAGTAAAGGTCAGGTGCTCCAAAGAGTCT | Amplifying MoEND3 3' flank sequence for gene knock out |
| CYFPEMC2F | CGACTCACTATAGGGCGAATTGGGTACTCAAATTGAGCTTGGACACAATAAGCGTGGC | Construction of *MoEMC2*-*CYFP* |
| CYFPEMC2R | GTTCGGGATCTTGCAGGCCGGGCGTTTTGGAGCGGAGGAATTGTCCTGG | Construction of *MoEMC2*-*CYFP* |
| NYFPEMC2R | CGACTCACTATAGGGCGAATTGGGTACTCAAATTGAGCTTGGACACAATAAGCGTGGC | Construction of *MoEMC2*-*NYFP* |
| NYFPEMC2R | GCTCACCATCGTGGCGATGGAGCGTTTTGGAGCGGAGGAATTGTCCTGG | Construction of *MoEMC2*-*NYFP* |
| SEMC2F | ACTCACTATAGGGCGAATTGGGTACTCAAATTGGTTAGCTTGGACACAATAAGCGTGG | Construction of *MoEMC2*-*S* |
| SEMC2R | TTCGAATTTAGCAGCAGCGGTTTCTTTTTTTGGAGCGGAGGAATTGTCCTGG | Construction of *MoEMC2*-*S* |
| GFPEMC2F | ACTCACTATAGGGCGAATTGGGTACTCAAATTGGTTAAGCGTGGCGATCTCCTCTGTGC | Construction of *MoEMC2*-*GFP* |
| GFPEMC2R | CACCACCCCGGTGAACAGCTCCTCGCCCTTGCTCACTTTTGGAGCGGAGGAATTGTCC | Construction of *MoEMC2*-*GFP* |
| ADEMC2F | CGCCATATGATGCCGCCCTCACTACTCCATCCAC | Construction of *pGADT7*-*MoEMC2* |
| ADEMC2R | CCGGAATTCCTATTTTGGAGCGGAGGAATTGTCC | Construction of *pGADT7*-*MoEMC2* |
| BDEMC2F | AAGCTGATCTCAGAGGAGGACCTGCATATGATGCCGCCCTCACTACTCCATCCAC | Construction of *pGBKT7*-*MoEMC2* |
| BDEMC2R | CGCTGCAGGTCGACGGATCCCCGGGAATTCCTATTTTGGAGCGGAGGAATTGTCC | Construction of *pGBKT7*-*MoEMC2* |
| GSTEMC2F | CCGGAATTCATGCCGCCCTCACTACTCCATCCAC | Construction of *pGEX4T*-*2*-*MoEMC2* |
| GSTEMC2R | CCGCTCGAGCTATTTTGGAGCGGAGGAATTGTCC | Construction of *pGEX4T*-*2*-*MoEMC2* |
| RFPEMC2F1 | ACTCACTATAGGGCGAATTGGGTACTCAAATTGGTTAAGCGTGGCGATCTCCTCTGTGC | Construction of *Mo EMC2*-*RFP* |
| RFPEMC2R1 | TGATGACGTCCTCGGAGGAGGCCATTTTTGGAGCGGAGGAATTGTCCTGG | Construction of *MoEMC2*-*RFP* |
| RFPEMC2F2 | CCAGGACAATTCCTCCGCTCCAAAAATGGCCTCCTCCGAGGACGTCATCA | Construction of *MoEMC2*-*RFP* |
| RFPEMC2R2 | GCGCAGAGGAGCGTGAATGTTGAGTGGAATGATGGCGCCGGTGGAGTGGCGGCCCTC | Construction of *MoEMC2*-*RFP* |
| BDCKA1F | AAGCTGATCTCAGAGGAGGACCTGCATATGATGCACAGCATGGCGCGCGTTTACG | Construction of *pGBKT7*-*MoCka1* |
| BDCKA1R | CGCTGCAGGTCGACGGATCCCCGGGAATTCTCAAGCCGAGGTGTTGGTTCCTGCA | Construction of *pGBKT7*-*MoCka1* |
| GSTCKA1F | CCGGAATTCATGCACAGCATGGCGCGCGTTTACG | Construction of *pGEX4T*-*2*-*MoCka1* |
| GSTCKA1R | ACGCGTCGACTCAAGCCGAGGTGTTGGTTCCTGCA | Construction of *pGEX4T*-*2*-*MoCka1* |
| SCKA1F | ACTCACTATAGGGCGAATTGGGTACTCAAATTGGTTTTGGTTGCTAAGGTTGGGCCTCC | Construction of *pXY203*-*MoCka1* |
| SCKA1R | TTCGAATTTAGCAGCAGCGGTTTCTTTAGCCGAGGTGTTGGTTCCTGCAGCA | Construction of *pXY203*-*MoCka1* |
| CYFPCKA1F | CGACTCACTATAGGGCGAATTGGGTACTCAAATTGTTGGTTGCTAAGGTTGGGCCTCC | Construction of *MoCka1*-*CYFP* |
| CYFPCKA1R | GTTCGGGATCTTGCAGGCCGGGCGAGCCGAGGTGTTGGTTCCTGCAGCA | Construction of *MoCka1*-*CYFP* |
| NGFPCKA1F1 | ACTCACTATAGGGCGAATTGGGTACTCAAATTGGTTTTGGTTGCTAAGGTTGGGCCTCC | Construction of *GFP*-*MoCka1* |
| NGFPCKA1R1 | AGCTCCTCGCCCTTGCTCACCATCTGGTGGTGCGGCAGCACCGAC | Construction of *GFP*-*MoCka1* |
| NGFPCKA1F2 | GTCGGTGCTGCCGCACCACCAGATGGTGAGCAAGGGCGAGGAGCT | Construction of *GFP*-*MoCka1* |
| NGFPCKA1R2 | AAACGCGCGCCATGCTGTGCATCTTGTACAGCTCGTCCATGC | Construction of *GFP*-*MoCKa1* |
| NGFPCKA1F3 | GCATGGACGAGCTGTACAAGATGCACAGCATGGCGCGCGTTT | Construction of *GFP*-*MoCka1* |
| NGFPCKA1R3 | GCGCAGAGGAGCGTGAATGTTGAGTGGAATGATTCAAGCCGAGGTGTTGGTTCCTGC | Construction of *GFP*-*MoCka1* |
| BDCKB1F | AAGCTGATCTCAGAGGAGGACCTGCATATGATGTCGACTTCGTCGGGAACGCCAG | Construction of *pGBKT7*-*MoCKB1* |
| BDCKB1R | CGCTGCAGGTCGACGGATCCCCGGGAATTCTCACAATTCCGACTCTGCCCCATTT | Construction of *pGBKT7*-*MoCKB1* |
| GSTCKB1F | TCCCCCGGGATGTCGACTTCGTCGGGAACGCCAG | Construction of *pGEX4T*-*2*-*MoCKB1* |
| GSTCKB1R | CCGCTCGAGCAATTCCGACTCTGCCCCATTTGTT | Construction of *pGEX4T*-*2*-*MoCKB1* |
| SCKB1F | ACTCACTATAGGGCGAATTGGGTACTCAAATTGGTTAACTAGGACCCTCGGTTACTTAC | Construction of *pXY203*-*MoCKB1* |
| SCKB1R | TTCGAATTTAGCAGCAGCGGTTTCTTT CAATTCCGACTCTGCCCCATTTGTT | Construction of *pXY203*-*MoCKB1* |
| CYFPCKB1  F | CGACTCACTATAGGGCGAATTGGGTACTCAAATTGAACTAGGACCCTCGGTTACTTAC | Construction of *MoCKB1*-*CYFP* |
| CYFPCKB1R | GTTCGGGATCTTGCAGGCCGGGCGCAATTCCGACTCTGCCCCATTTGTT | Construction of *MoCKB1*-*CYFP* |
| NGFPCKB1  F1 | ACTCACTATAGGGCGAATTGGGTACTCAAATTGGTTAACTAGGACCCTCGGTTACTTA | Construction of *GFP*-*MoCKB1* |
| NGFPCKB1R1 | AACAGCTCCTCGCCCTTGCTCACCATTGCGACCGAGAAGAACGCGCTT | Construction of *GFP*-*MoCKB1* |
| NGFPCKB1  F2 | AAGCGCGTTCTTCTCGGTCGCAATGGTGAGCAAGGGCGAGGAGCTGTT | Construction of *GFP*-*MoCKB1* |
| NGFPCKB1R2 | CGTTCCCGACGAAGTCGACATCTTGTACAGCTCGTCCATGC | Construction of *GFP*-*MoCKB1* |
| NGFPCKB1  F3 | GCATGGACGAGCTGTACAAGATGTCGACTTCGTCGGGAACG | Construction of *GFP*-*MoCKB1* |
| NGFPCKB1R3 | GCGCAGAGGAGCGTGAATGTTGAGTGGAATGATTCACAATTCCGACTCTGCCCCATTT | Construction of *GFP*-*MoCKB1* |
| BDCKB2F | AAGCTGATCTCAGAGGAGGACCTGCATATGATGGAAGACTTTGGCAGCGAGTCGG | Construction of *pGBKT7*-*MoCKB2* |
| BDCKB2R | CGCTGCAGGTCGACGGATCCCCGGGAATTCTCAGACACCTTGCATCATGCTATCC | Construction of *pGBKT7*-*MoCKB2* |
| GSTCKB2F | CCGGAATTCATGGAAGACTTTGGCAGCGAGTCGG | Construction of *pGEX4T*-*2*-*MoCKB2* |
| GSTCKB2R | CCGCTCGAGGACACCTTGCATCATGCTATCCCCA | Construction of *pGEX4T*-*2*-*MoCKB2* |
| SCKB2F | ACTCACTATAGGGCGAATTGGGTACTCAAATTGGTTATAACTTTAAACCATTGGCGGTT | Construction of *pXY203*-*MoCKB2* |
| SCKB2R | TTCGAATTTAGCAGCAGCGGTTTCTTT GACACCTTGCATCATGCTATCCCCA | Construction of *pXY203*-*MoCKB2* |
| CYFPCKB2  F | CGACTCACTATAGGGCGAATTGGGTACTCAAATTGATAACTTTAAACCATTGGCGGTTG | Construction of *MoCKB2*-*CYFP* |
| CYFPCKB2R | GTTCGGGATCTTGCAGGCCGGGCGGACACCTTGCATCATGCTATCCCCA | Construction of *MoCKB2*-*CYFP* |
| NGFPCKB2  F1 | ACTCACTATAGGGCGAATTGGGTACTCAAATTGGTTATAACTTTAAACCATTGGCGGTT | Construction of *GFP*-*MoCKB2* |
| NGFPCKB2R1 | CTCCTCGCCCTTGCTCACCATAATGGCAGCGACCTTTGGTGGGA | Construction of *GFP*-*MoCKB2* |
| NGFPCKB2  F2 | TCCCACCAAAGGTCGCTGCCATTATGGTGAGCAAGGGCGAGGAG | Construction of *GFP*-*MoCKB2* |
| NGFPCKB2R2 | ACTCGCTGCCAAAGTCTTCCATCTTGTACAGCTCGTCCATGCCG | Construction of *GFP*-*MoCKB2* |
| NGFPCKB2  F3 | CGGCATGGACGAGCTGTACAAGATGGAAGACTTTGGCAGCGAGT | Construction of *GFP*-*MoCKB2* |
| NGFPCKB2R3 | GCGCAGAGGAGCGTGAATGTTGAGTGGAATGATTCAGACACCTTGCATCATGCTA | Construction of *GFP*-*MoCKB2* |
| HISMAGAF1 | GCCATGGCTGATATCGGATCCGAATTCATGATGGGCGCTTGCATGAGTGCGA | Construction of *HIS*-*MoCKB2* |
| HISMAGAR1 | GTGGTGCTCGAGTGCGGCCGCAAGCTTTTACAGAATACCTGAGTCCTTCAGG | Construction of *HIS*-*MoCKB2* |
| RFPMAGAF1 | ACTCACTATAGGGCGAATTGGGTACTCAAATTGGTTAGTGAGGAACATGTCAAGGCAG | Construction of *GFP*-*MoMAGA* |
| RFPMAGAR1 | ATGACGTCCTCGGAGGAGGCCATCTCGACCATGTAGTCGAGCAGATAG | Construction of *GFP*-*MoMAGA* |
| RFPMAGAF2 | CTATCTGCTCGACTACATGGTCGAGATGGCCTCCTCCGAGGACGTCAT | Construction of *GFP*-*MoMAGA* |
| RFPMAGAR2 | GGTCTATCTCTGCCTGTGGTCCCGAGGCGCCGGTGGAGTGGCGGCCCT | Construction of *GFP*-*MoMAGA* |
| RFPMAGAF3 | AGGGCCGCCACTCCACCGGCGCCTCGGGACCACAGGCAGAGATAGACC | Construction of *GFP*-*MoMAGA* |
| RFPMAGAR3 | GCGCAGAGGAGCGTGAATGTTGAGTGGATGATTTACAGAATACCTGAGTCCTTCAGG | Construction of *GFP*-*MoMAGA* |
