# Supplementary figures and images for: The rice blast fungus MoRgs1 functioning in cAMP signaling and pathogenicity is regulated by casein kinase MoCk2 phosphorylation and modulated by membrane protein MoEmc2
Source: PLoS Pathog. 2021 Jun 16;17(6):e1009657. doi: 10.1371/journal.ppat.1009657 (PMC8208561; doi:10.1371/journal.ppat.1009657)

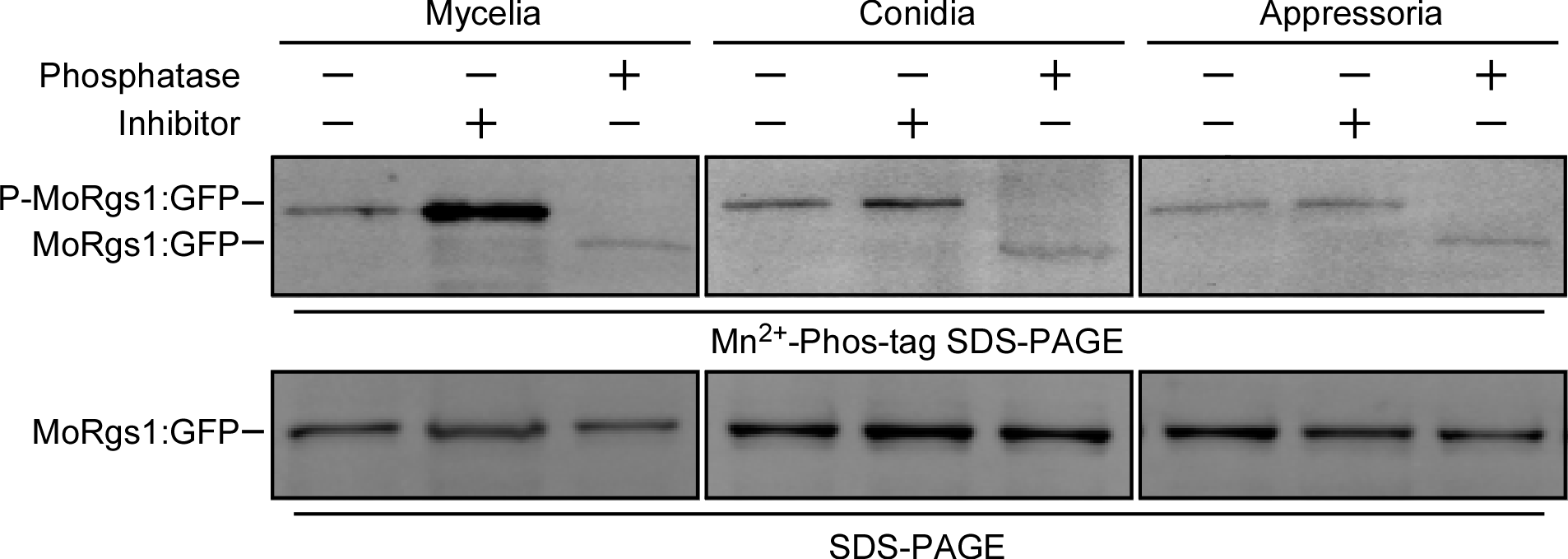

Supplement: S1 Fig — MoRgs1-GFP proteins were extracted from transformants at mycelia, conidia, and appressoria stages then treated with phosphatase and phosphatase inhibitors. Mn2+-Phos-tag SDS-PAGE and normal SDS-PAGE were used to conduct Western blot analysis with the anti-GFP antibody. The extent of MoRgs1 phosphorylation was estimated by the mobility shift assay. (TIF) [file ppat.1009657.s002.tif]

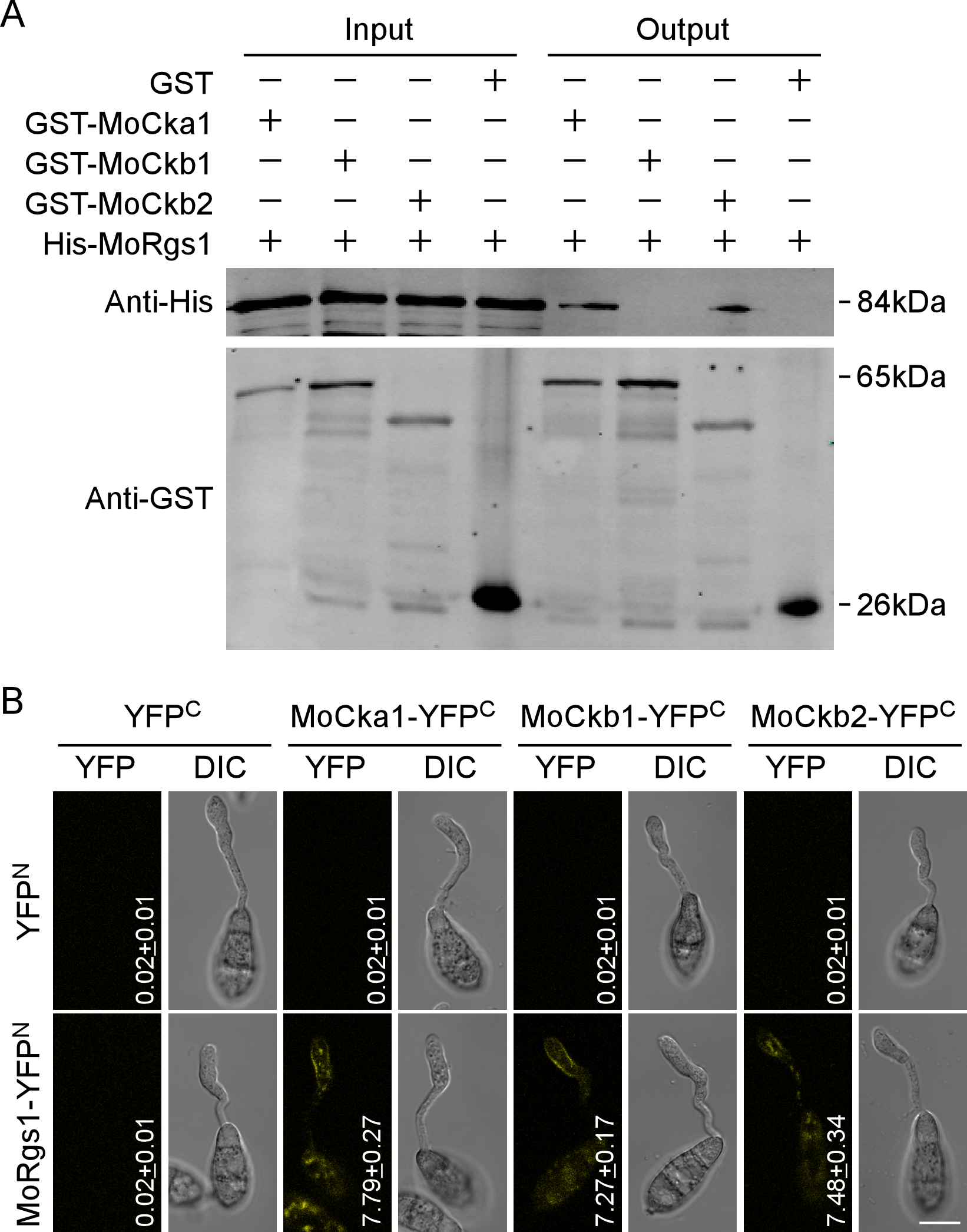

Supplement: S2 Fig — (A) The interaction among all three subunits of MoCk2 holoenzyme (GST-MoCka1, GST-MoCkb1, and GST-MoCkb2) with His-MoRgs1 were conducted by GST pull-down assays. GST-MoCka1, GST-MoCkb1, GST-MoCkb2, His-MoRgs1, and GST were expressed and purified by affinity chromatography. Bound proteins were separated by SDS-PAGE in duplicate and analyzed by Western blot with the anti-His (Mouse; M20001; Abmart) and anti-GST antibodies (Mouse; M20007; Abmart). (B) The interaction among all three subunits of MoCk2 holoenzyme (MoCka1-YFPC, MoCkb1-YFPC, and MoCkb2-YFPC) with MoRgs1-YFPN were conducted by BiFC. Empty YFPC and empty YFPN constructs were used as a negative control. The co-transformants were observed at the germ tube hooking stage (3 h) with laser scanning microscopy (Zeiss LSM710 laser scanning microscope, 63 × oil, Bar = 10 μm). The mean and standard deviation of fluorescence intensity were denoted over 50 germinated conidia that were randomly chosen. (TIF) [file ppat.1009657.s003.tif]

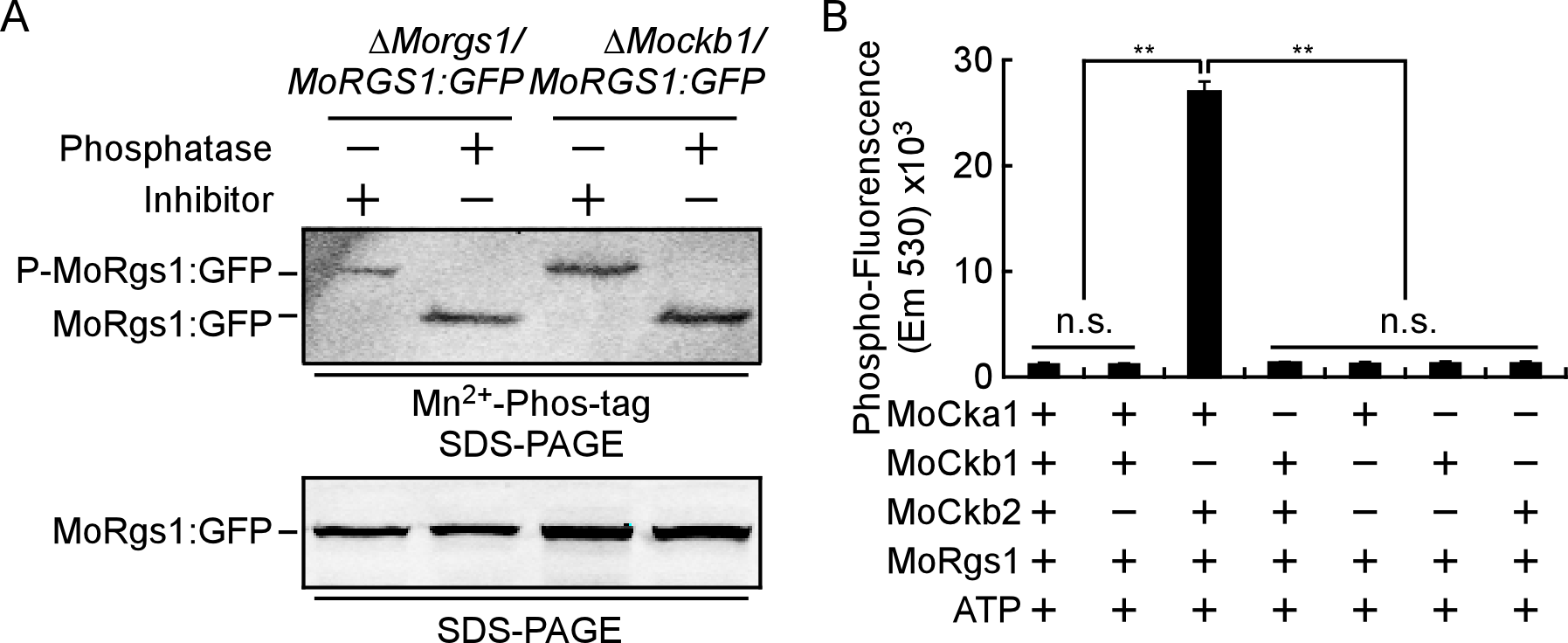

Supplement: S3 Fig — (A) Phosphorylation analysis of MoRgs1 in vivo. Total proteins treated with phosphatase and phosphatase inhibitors were detected by the GFP antibody. Bands were shifted by Mn2+-Phos-tag SDS-PAGE and normal SDS-PAGE, respectively. (B) Phosphorylation analysis in vitro by the fluorescence detection in tube (FDIT) method. Purified proteins of GST-MoCka1, GST-MoCkb1, GST-MoCkb2, and His-MoRgs1 were constructed for the protein kinase reaction in the presence of ATP. Fluorescence was measured in a microplate reader (**P < 0.01, n = 3). (TIF) [file ppat.1009657.s004.tif]

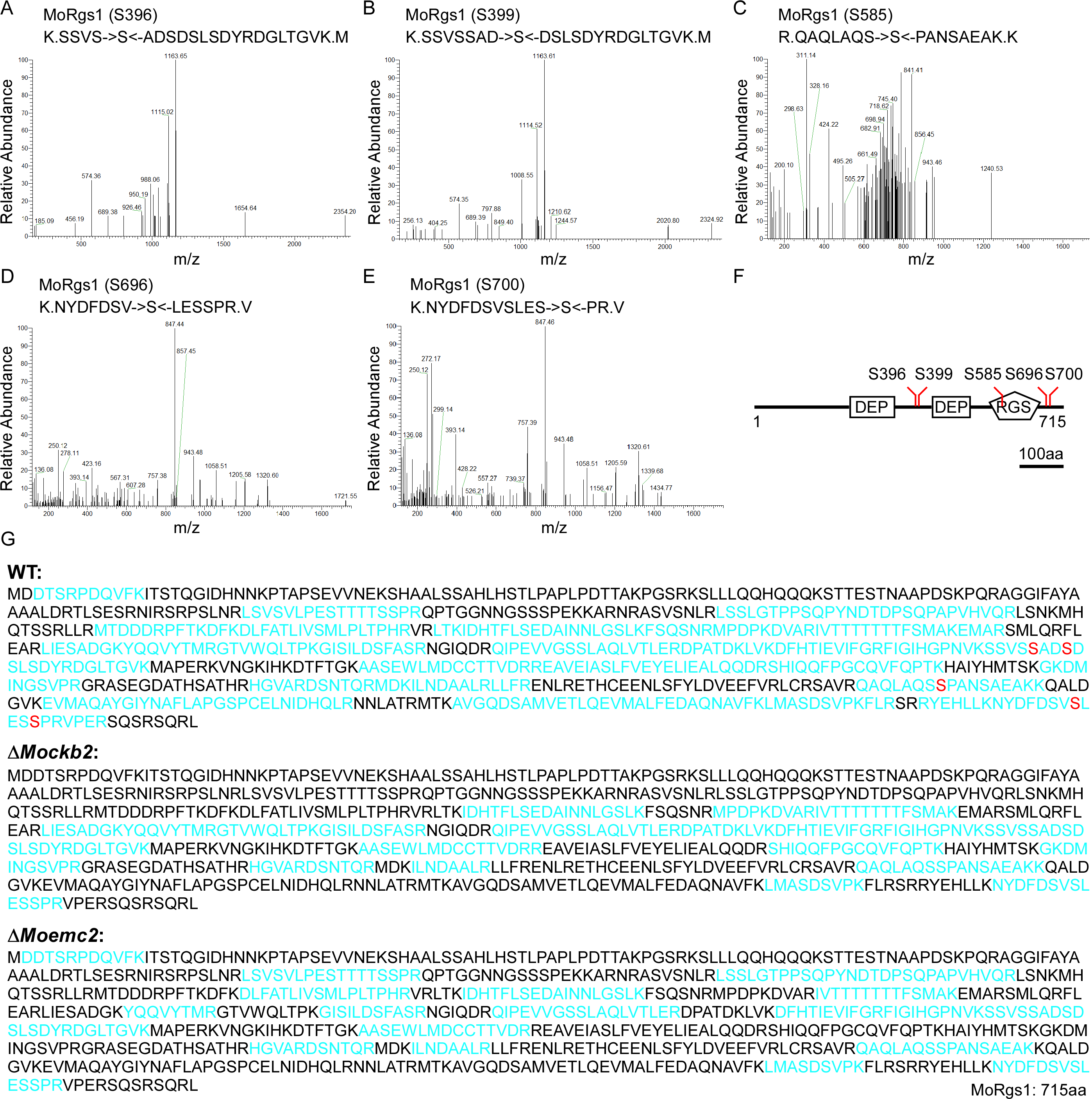

Supplement: S4 Fig — (A-E) Identified differentiated phosphorylation sites of MoRgs1 between the WT and ΔMockb2 strains by LC-MS-MS (Q-E). The five phosphorylation sites (S396, S399, S585, S696, and S700) were identified in the wild type strain. Phosphorylation site sand sequences are annotated in the upper panel. (F) A model of five serine sites located at the MoRgs1 domains. (G) Covered peptides and phosphorylated sites in the wild type, ΔMockb2, and ΔMoemc2 strains. Colorful letters represent the amino acid sequences covered by mass spectrometry. Red letters represent phosphorylation sites newly identified. (TIF) [file ppat.1009657.s005.tif]

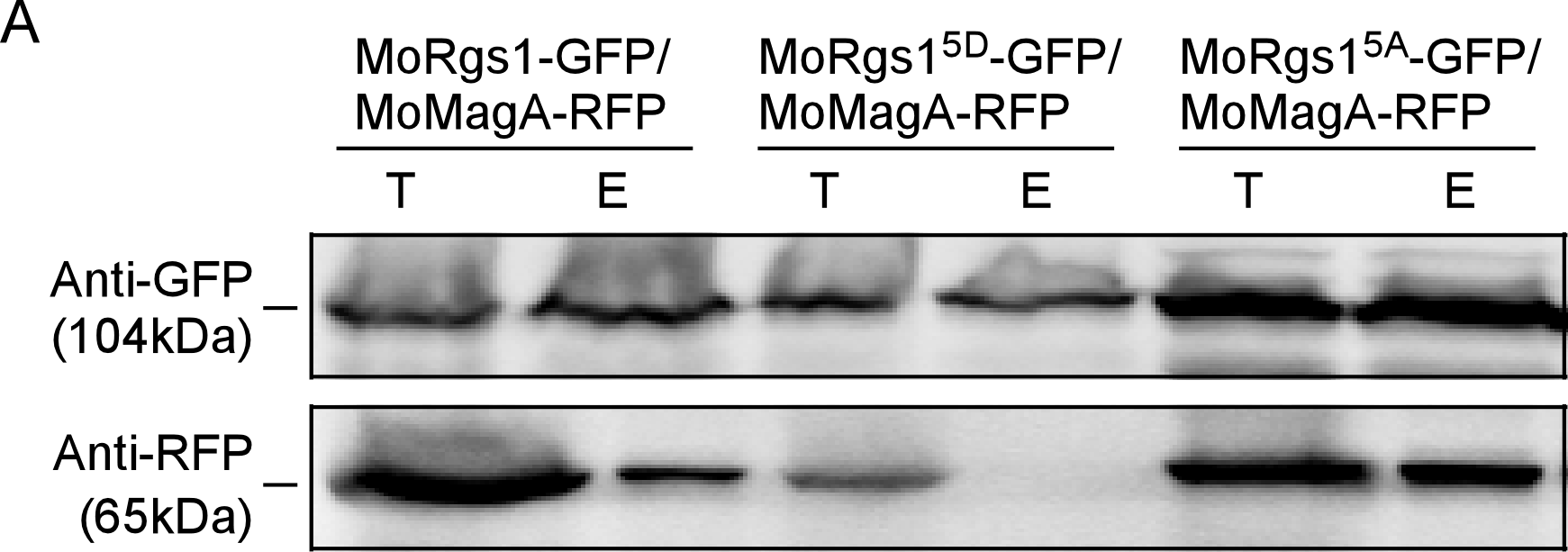

Supplement: S5 Fig — Co-IP analysis for the interaction between MoMagA and MoRgs1, MoRgs15A, and MoRgs15D, respectively. Total proteins were extracted and incubated with the anti-GFP agarose and then eluted for Western blot analysis using anti-RFP or anti-GFP antibodies. (TIF) [file ppat.1009657.s006.tif]

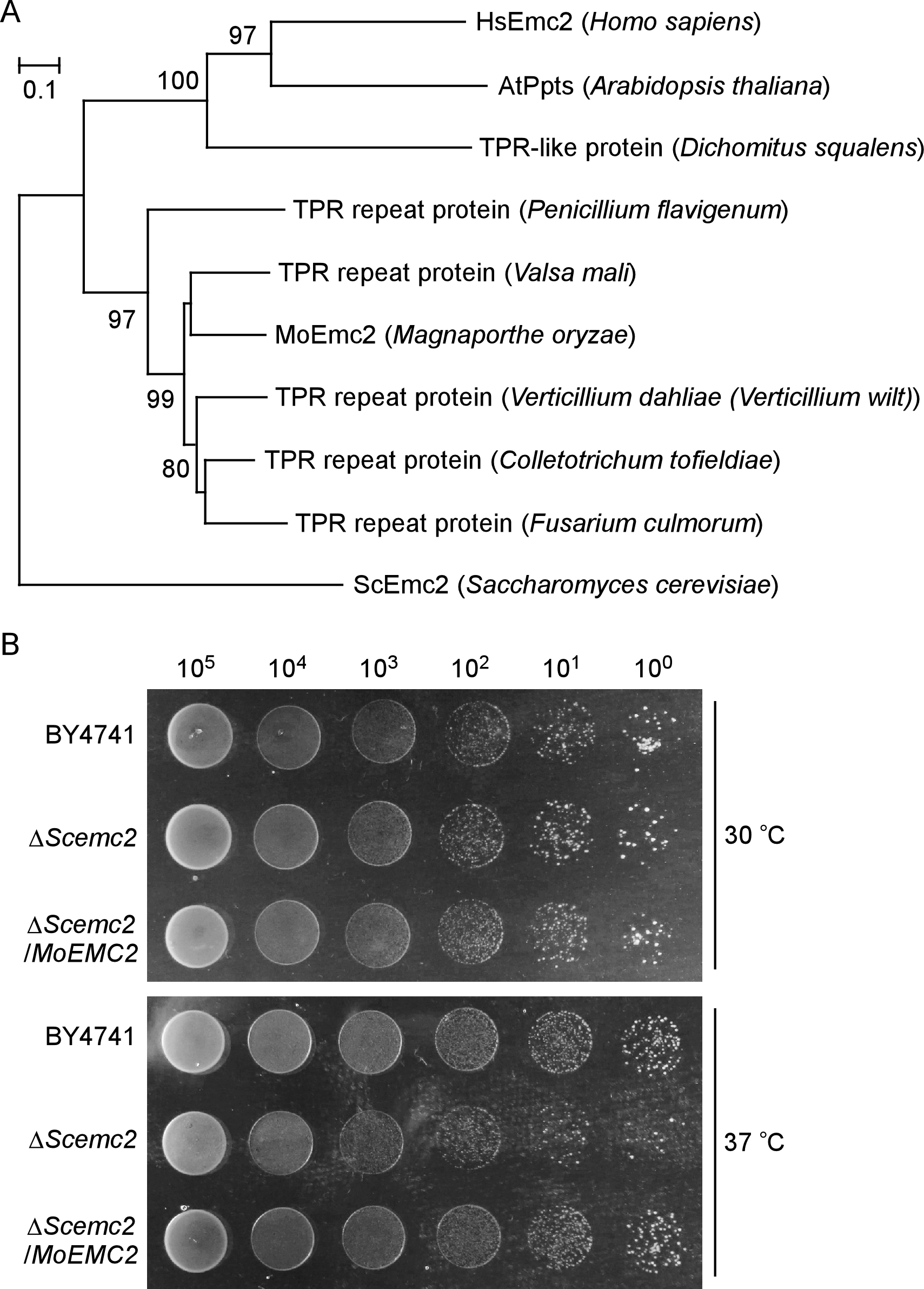

Supplement: S6 Fig — (A) The amino acid sequences of diverse Emc2 proteins from corresponding organisms were aligned using the CLUSTAL_W. The neighbor-joining tree was constructed by MEGA 7.0 with 1000 bootstrap replicates. GenBank accession numbers and the corresponding species names are as listed: XP_003711387.1 (Magnaporthe oryzae MoEmc2), NP_012621.1 (Saccharomyces cerevisiae ScEmc2), KUI71153.1 (Valsa mali TPR repeat protein), PTD09165.1 (Fusarium culmorum TPR repeat protein), KZL69988.1 (Colletotrichum tofieldiae TPR repeat protein), XP_009648592.1 (Verticillium dahliae TPR repeat protein), OQE20945.1 (Penicillium flavigenum TPR repeat protein), TBU37051.1 (Dichomitus squalens TPR-like protein), NP_850995.1 (Arabidopsis thaliana AtPpts), and NP_055488.1 (Homo sapiens HsEmc2). (B) MoEMC2 suppressed the heat sensitivity of the yeast Δemc2 strain. 10-fold serial dilutions of BY4741, ΔScemc2, and ΔScemc2 transformed with pYES2-MoEMC2 constructs were grown on SD-Met-Leu-His-Ura (galactose) plates at 30°C and 37°C for 4 days and then photographed. (TIF) [file ppat.1009657.s007.tif]

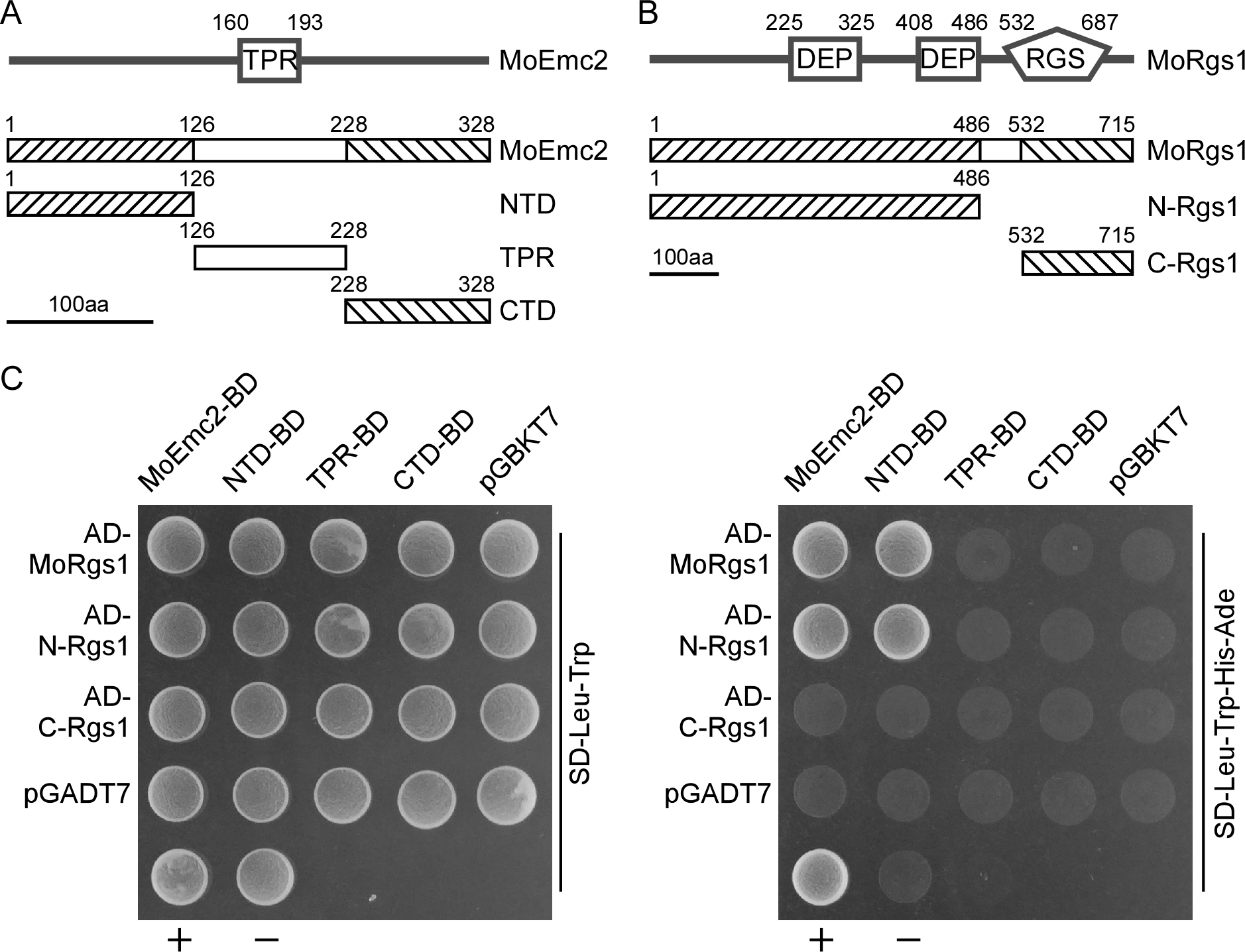

Supplement: S7 Fig — (A) Structure and domain prediction of MoEmc2 using SMART (http://smart.embl-heidelberg.de/). The positions of the domains within the proteins were indicated by amino acid numbers. The full length of MoEMC2 was divided into NTD, TPR, and CTD domains before being ligated in pGBKT7. (B) MoRgs1 has two DEP domains at the N-terminus and one RGS domain at the C-terminus [22, 31]. Similar methods were used to conduct the following MoRgs1 vectors in pGADT7: AD-MoRgs1, AD-N-Rgs1, and AD-C-Rgs1. (C) The full length and regions of MoRgs1 and MoEmc2 were assayed by Y2H. The yeast co-transformants expressing the bait and prey constructs were isolated on the SD-Leu-Trp plate for 3 d and screened by SD-Ade-His-Leu-Trp plates for 5 d. (TIF) [file ppat.1009657.s008.tif]

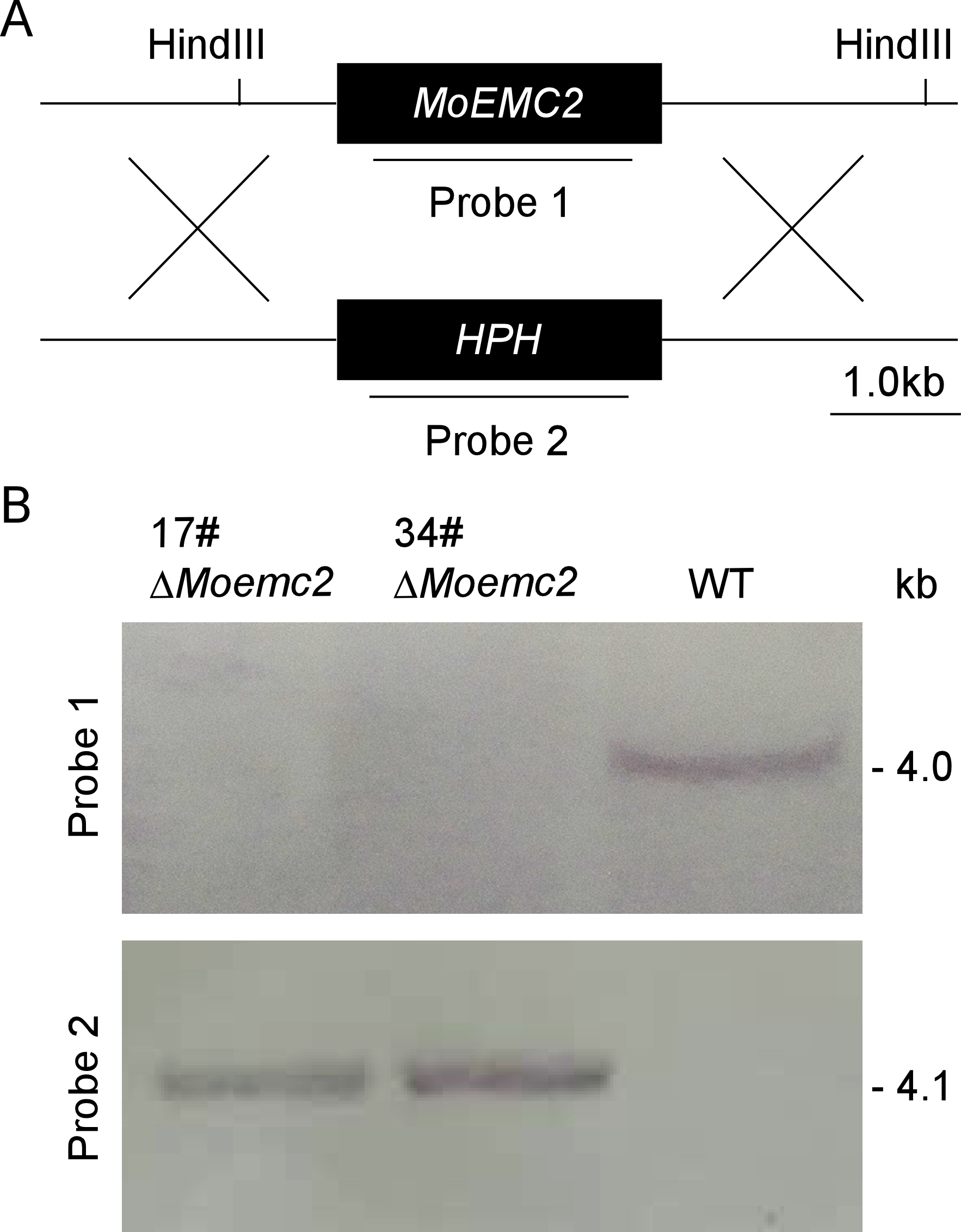

Supplement: S8 Fig — (A) A model of the MoEMC2 gene deletion by homologous recombination in M. oryzae. (B) Gene-specific probe (probe 1) and hygromycin phosphotransferase (HPH) probe (probe2) were used in Southern hybridization. Thick square frames indicate the sites of MoEMC2 and HPH genes. Thin lines below the square frames indicate sequence-specific gene probes. (TIF) [file ppat.1009657.s009.tif]

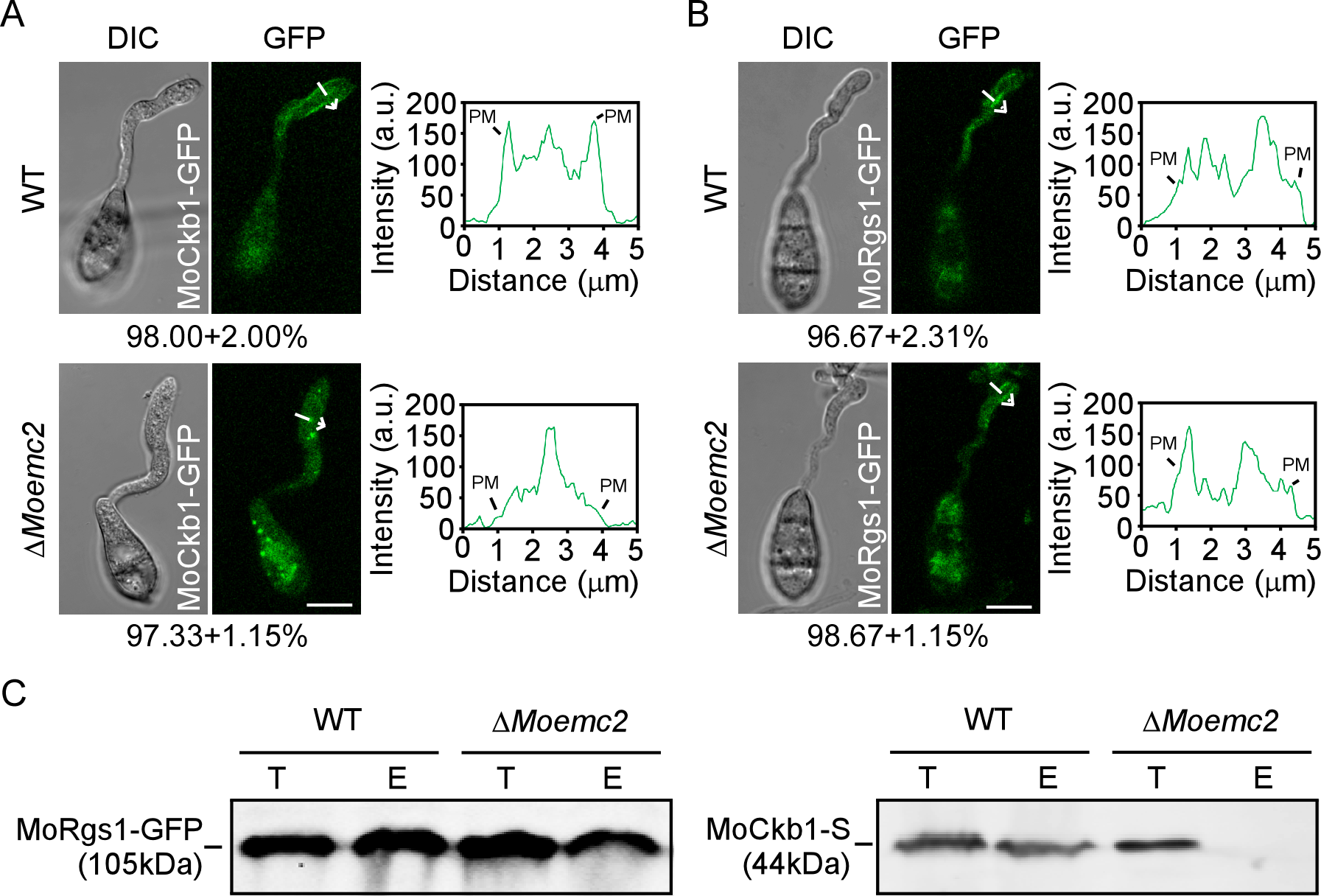

Supplement: S9 Fig — (A and B) Fluorescence GFP labeled MoCkb1-GFP, and MoRgs1-GFP fusion constructs were introduced into the WT and ΔMoemc2 strains at the germ tube hooking stage (3 hpi). Insets highlight areas analyzed by line-scan. Bar = 10 μm. Percentage of a pattern showed in image was calculated by observation for 50 germinated conidia that were randomly chosen, and observation was conducted for 3 times. (C) Co-IP assays for the interaction between MoRgs1-GFP with MoCkb1-S in the WT and ΔMoemc2 strains. Total proteins were extracted and eluted from the anti-GFP agarose beads before being analyzed by immunoblotting with corresponding antibodies. T: Total protein E: Elution. (TIF) [file ppat.1009657.s010.tif]

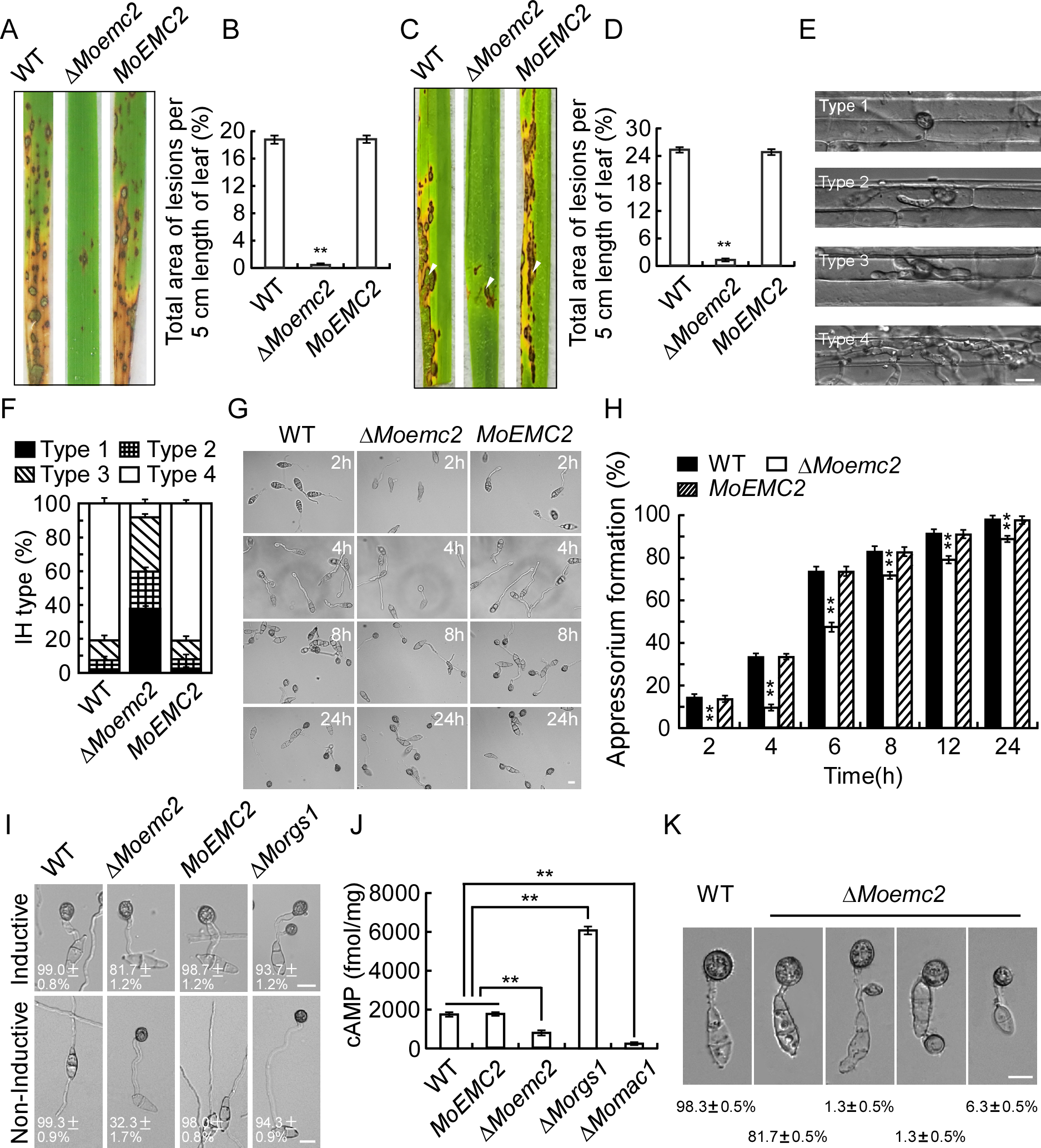

Supplement: S10 Fig — (A and B) Rice spraying assays and lesion area statistics. Conidial suspensions (5 × 10^4^ spores/ml) were sprayed onto 2 week-old rice seedlings (CO-39). Diseased rice leaves were photographed and percentages of per 5 cm length leaf lesion area were analyzed by ImageJ after 7 days of inoculation. Values are means of three replications and SD (**P < 0.01, n = 10). (C and D) Rice sheath injecting assays in vivo and lesion area statistics. Conidial suspensions (2 × 10^5^ spores/ml) were sprayed onto 4 week-old rice seedlings (CO-39). Diseased rice leaves were photographed and percentages per 5 cm length leaf lesion area were analyzed by ImageJ after 5 days of inoculation. Values are means of three replications and SD (**P < 0.01, n = 10). White triangles point out the injection sites. (E and F) Rice sheath injecting assays in vitro and classification statistics. Invasive hyphae (IH, n = 100) in rice cells were observed at 36 hpi and 4 types of were quantified and statistically analyzed. Error bars represent SD from three independent replicates. (G and H) Appressorium formation assays and statistics analysis. Conidia of the WT, ΔMoemc2 and complemented ΔMoemc2 (ΔMoemc2/MoEMC2) strains were dropped on hydrophobic surfaces and the dynamics of appressorium formation were photographed at various times (**P < 0.01, n = 100). Bar = 10 μm. (I) Appressorium formation was assayed on hydrophobic (the upper panel) and hydrophilic (the upper panel) surfaces for 24 hpi. Percentages of Mean and SD were shown at the lower panel. (J) Intracellular cAMP levels in the mycelia of the indicated strains cultured for 2 d in CM were quantified by HPLC (**P < 0.01, n = 3). (K) Morphological characteristics of the WT and ΔMoemc2 strains. Percentages of Mean and SD were depicted at the lower panel (**P < 0.01, n = 100). Bar = 10 μm. (TIF) [file ppat.1009657.s011.tif]

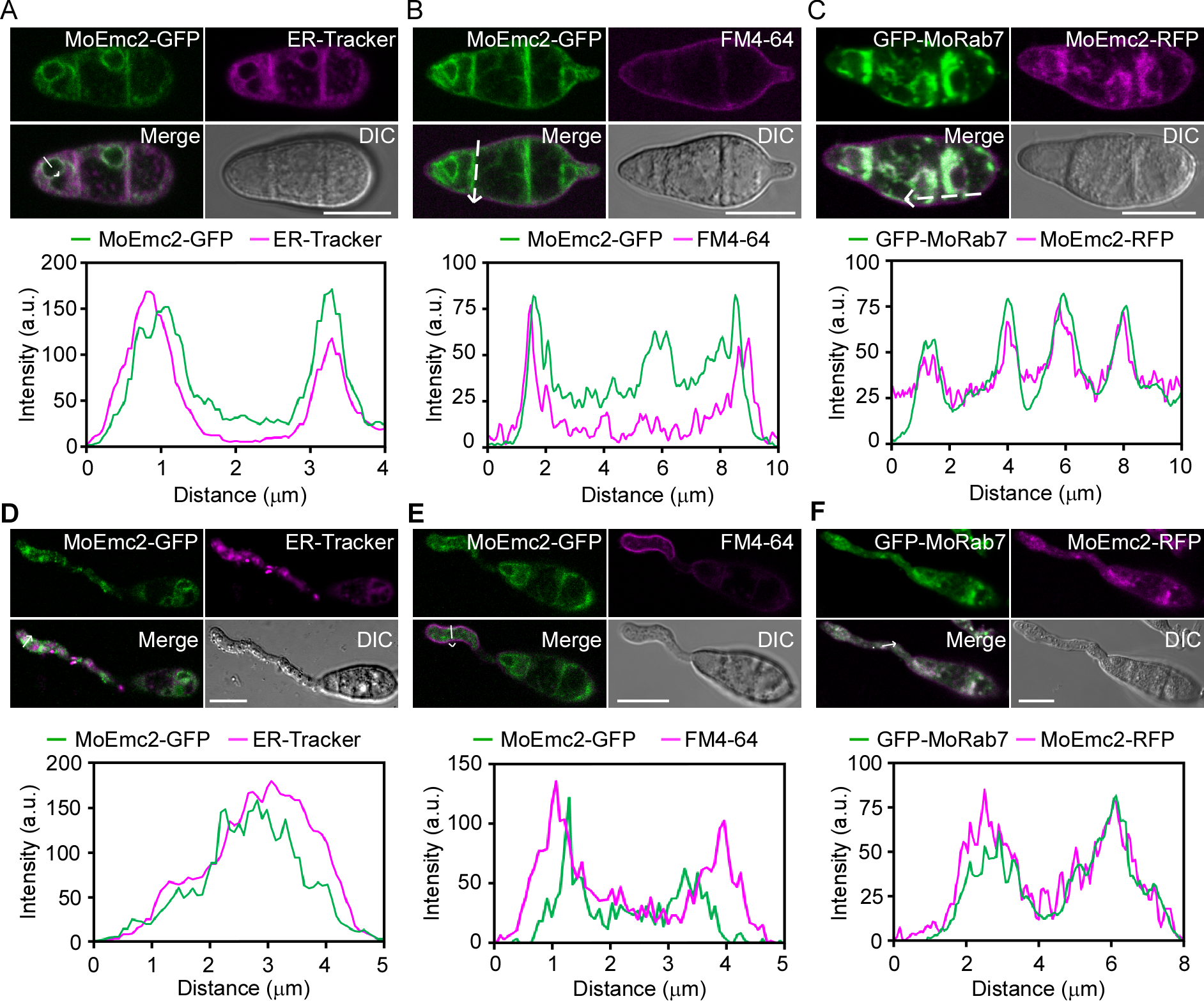

Supplement: S11 Fig — (A and D) MoEmc2-GFP transformants were stained by endoplasmic reticulum dye ER-Tracker at conidia and germ tube hook stages. (B and E) MoEmc2-GFP transformants were stained by FM4-64 at the conidia and germ tube hooking stages (3 h). (C and F) Late endosome marker GFP-MoRab7 was co-transformed with MoEmc2-RFP in the WT strain and observation was made at the conidia and germ tube hooking stages. All assays were observed 100 samples with three replicates and insets highlight areas analyzed by line-scan. Bar = 10 μm. (TIF) [file ppat.1009657.s012.tif]

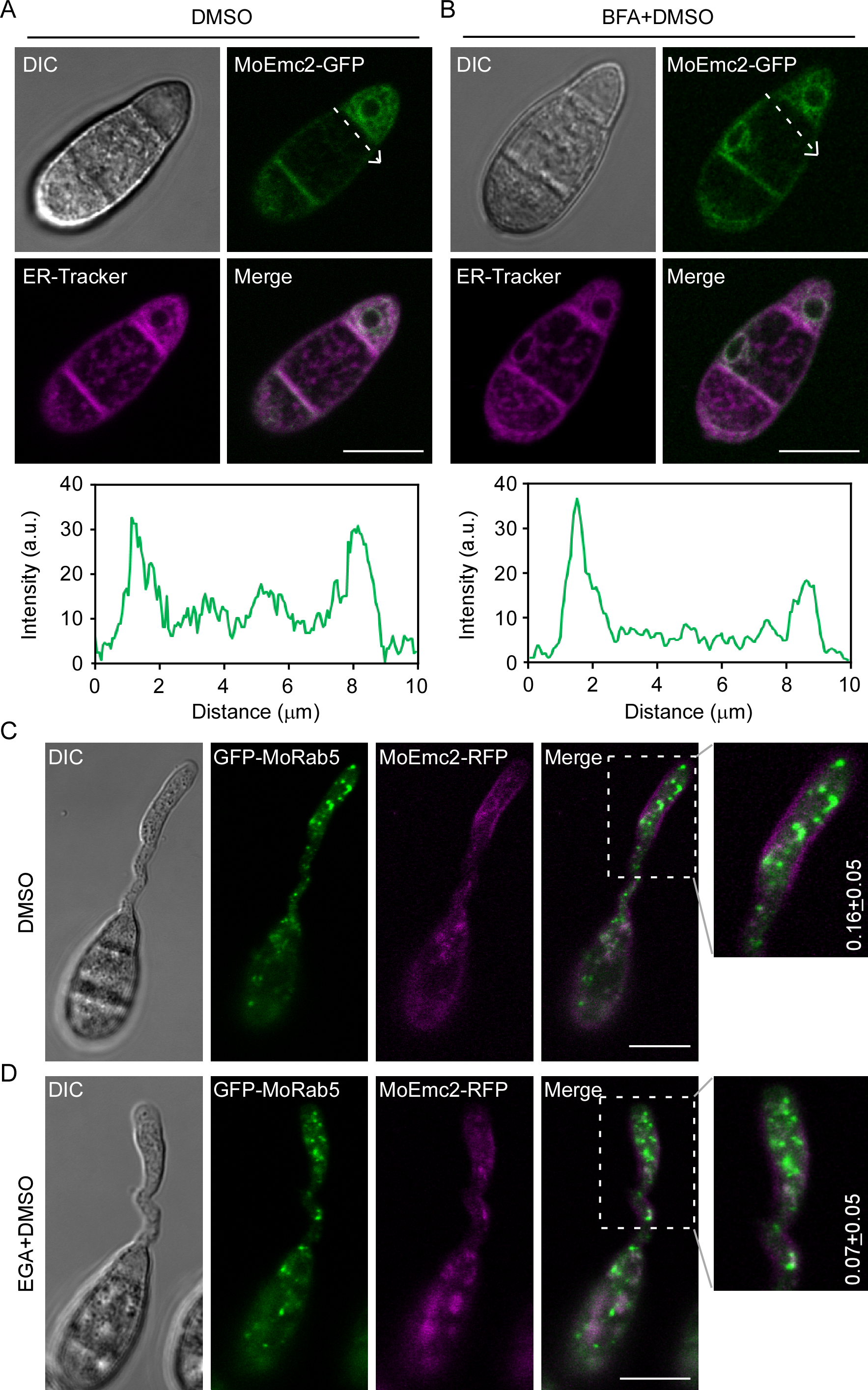

Supplement: S12 Fig — (A and B) ER-Golgi trafficking inhibitor BFA was used in conidium assays with DMSO solvent as control. Insets highlight areas analyzed by line-scan. Bars = 10 μm. (C and D) Early to late endosome inhibitor EGA assay was conducted in GFP-MoRab5 and MoEmc2-RFP co-transformants (3 h, germ tube hooking stage). Percentages of the pattern (shown in the images) were calculated by the observation of 100 randomly chosen germinated conidia, and the observation was conducted 3 times. The extent of fluorescence overlap was estimated with the Pearson correlation coefficient calculated by ImageJ coloc2. Mean and standard deviation were indicated in the right column (n = 30). Bars = 10 μm. (TIF) [file ppat.1009657.s013.tif]
