# Supplementary material for: The rice blast fungus MoRgs1 functioning in cAMP signaling and pathogenicity is regulated by casein kinase MoCk2 phosphorylation and modulated by membrane protein MoEmc2
Source: PLoS Pathog. 2021 Jun 16;17(6):e1009657. doi: 10.1371/journal.ppat.1009657 (PMC8208561; doi:10.1371/journal.ppat.1009657)
Supplement: S1 Text — The bait construct AD-MoRgs1 was used to screen a yeast two-hybrid cDNA library constructed with an RNA pool from various stages, including conidia and infectious hyphae (0, 2, 4, 8, 12 and 24 h). (DOCX) [file ppat.1009657.s014.docx]

S1 Text. Identification of MoRgs1 binding proteins.

| Gene locus | Protein name | Gene locus | Protein name |
| --- | --- | --- | --- |
| MGG_07480 | Tetratricopeptide repeat domain-containing protein | MGG_09820 | Hypothetical protein |
| MGG_07208 | Hypothetical protein | MGG_02517 | Hypothetical protein |
| MGG_17861 | Hypothetical protein | MGG_11701 | Hypothetical protein |
| MGG_14219 | Ubiquitin thioesterase | MGG_04847 | Peptidase M14 |
| MGG_03241 | Arrestin domain-containing protein | MGG_00461 | Hypothetical protein |
| MGG_05651 | Casein kinase II subunit beta-2 | MGG_00396 | PaaI_thioesterase |
| MGG_06952 | Ribosomal protein L38e | MGG_06481 | TRNA pseudouridine synthase D |
| MGG_00471 | Exocyst complex component Sec15 | MGG_00856 | Hypothetical protein |
| MGG_13836 | Hypothetical protein | MGG_11337 | Hypothetical protein |
| MGG_02823 | Beige/BEACH domain-containing protein | MGG_15739 | Inorganic pyrophosphatase |
| MGG_03857 | T-complex protein 1 subunit zeta | MGG_11784 | Hypothetical protein |
| MGG_06447 | SNF2 family snf21 | MGG_02479 | Nuclear transport factor 2 |
| MGG_06529 | Hypothetical protein | MGG_01725 | Deoxyhypusine hydroxylase |
| MGG_01732 | 3-carboxymuconate cyclase | MGG_12726 | Phenazine biosynthesis protein PhzF family protein |
| MGG_11554 | Seed imbibition protein | MGG_03372 | 40S ribosomal protein S20 |
| MGG_02714 | Assembly factor CBP4 | MGG_06930 | Hypothetical protein |
| MGG_11889 | T-complex protein 1 subunit epsilon | MGG_07038 | Hypothetical protein |
| MGG_10447 | Peptidyl-prolyl cis-trans isomerase | MGG_06726 | Septin like spn2 |
| MGG_02224 | Hypothetical protein | MGG_03186 | 1,4-alpha-glucan-branching enzyme |
