# Supplementary material for: The rice blast fungus MoRgs1 functioning in cAMP signaling and pathogenicity is regulated by casein kinase MoCk2 phosphorylation and modulated by membrane protein MoEmc2
Source: PLoS Pathog. 2021 Jun 16;17(6):e1009657. doi: 10.1371/journal.ppat.1009657 (PMC8208561; doi:10.1371/journal.ppat.1009657)
Supplement: S2 Text — a. Colony diameter of the indicated strains on CM and SDC media after 7 days incubation at 28°C. b. Dry weight of hyphal at 2 days after incubation in liquid complete medium at room temperature by shaken at 160 rpm. c. Quantification of the conidial production of the indicated strains formed on SDC cultures in the dark for 7 d, followed by incubation under constant illumination for 3 d at room temperature. d. Percentage of appressoria penetrating rice sheath epidermal cells at 24 h post-inoculation. All different capital letters in column show significant difference (Duncan’s new multiple range test,P < 0.01). All experiments were conducted with three biological repetitions and three replicates, mean and standard deviations were calculated. 5A: S396A S399A S585A S696A S700A 5D: S396D S399D S585D S696D S700D. (DOCX) [file ppat.1009657.s015.docx]

S2 Text. Phenotype analysis of the wild type, *∆Morgs1* mutant, site-directed mutagenesis mutants, complement strains.

|  | **Growth rate^a^ (cm)** | | **Biomass^b^** | **Conidiation^c^** | **Penetration^d^** |
| --- | --- | --- | --- | --- | --- |
| **Strain** | **CM** | **SDC** | **(mg)** | **(×100/cm^2^)** | **(%)** |
| WT | 4.60 ± 0.10^A^ | 4.00 ± 0.10^A^ | 0.1293 ± 0.0025^A^ | 397.7 ± 1.8^A^ | 78 ± 1.7^A^ |
| Δ*Morgs1* | 3.83 ± 0.06^C^ | 3.10 ± 0.10^C^ | 0.0587 ± 0.0030^B^ | 21.6 ± 1.1^C^ | 53 ± 1.5^C^ |
| Δ*Morgs1/MoRGS1*^5A^ | 3.90 ± 0.10^C^ | 3.13 ± 0.06^C^ | 0.0577 ± 0.0006^B^ | 20.1 ± 1.4^C^ | 52 ± 1.0^C^ |
| Δ*Morgs1/MoRGS1*^5D^ | 4.20 ± 0.10^B^ | 3.37 ± 0.06^B^ | 0.0587 ± 0.0015^B^ | 34.8 ± 1.3^B^ | 62 ± 2.0^B^ |
| Δ*Morgs1/MoRGS1* | 4.57 ± 0.06^A^ | 3.97 ± 0.06^A^ | 0.1280 ± 0.0030^A^ | 399.1 ± 0.2^A^ | 78 ± 1.5^A^ |
