# Supplementary material for: The rice blast fungus MoRgs1 functioning in cAMP signaling and pathogenicity is regulated by casein kinase MoCk2 phosphorylation and modulated by membrane protein MoEmc2
Source: PLoS Pathog. 2021 Jun 16;17(6):e1009657. doi: 10.1371/journal.ppat.1009657 (PMC8208561; doi:10.1371/journal.ppat.1009657)
Supplement: S3 Text — a. Colony diameter of the indicated strains on CM, OM, MM and SDC agar plates after 7 dark days incubation at 28°C. b. Dry weight of hyphae 2 days after incubation in liquid complete medium at 28°C with 160 rpm. c. Statistics of conidial production of the indicated strains growing 7 dark days on SDC agar plates followed by constant illumination (wavelength of 365 nm) for 3 d at room temperature. d. Percentage of appressorium formation on hydrophobic surface with 24 h post-inoculation. e. Percentage of collapsed appressorium treated with 1 M, 2 M, 3 M and 4 M glycerol solution for 5 min after appressorium formation with 24 hpi. All different capital letters in column show significant difference (Duncan’s new multiple range test, P < 0.01). All experiments were conducted with three biological repetitions and three replicates, mean and standard deviations were calculated. (DOCX) [file ppat.1009657.s016.docx]

S3 Text. Phenotype analysis of the wild type (WT), Δ*Moemc2* mutant, Δ*Moemc2/MoRGS1*^5D^ and complement (Δ*Moemc2/MoEMC2*) strains.

|  | **Growth rate^a^ (cm)** | | | | **Biomass^b^**  **(mg)** | **Conidiation^c^**  **(×10^3^/cm^2^)** | **Penetration^d^**  **(%)** | **Collapsed appressorium rate^e^ (%)** | | | |
| --- | --- | --- | --- | --- | --- | --- | --- | --- | --- | --- | --- |
| **Strain** | **CM** | **OM** | **MM** | **SDC** |  |  |  | **1 M** | **2 M** | **3 M** | **4 M** |
| WT | 4.4 ± 0.1^A^ | 3.8 ± 0.1^A^ | 3.6 ± 0.1^A^ | 3.6 ± 0.1^A^ | 0.1295 ± 0.0005^A^ | 62.03 ± 1.6^A^ | 78.3 ± 1.5^A^ | 9.3±1.5^C^ | 19.7±1.5^C^ | 62.3±0.6^C^ | 70.7±1.5^C^ |
| Δ*Moemc2* | 3.7 ± 0.1^C^ | 2.7 ± 0.1^C^ | 2.8 ± 0.1^C^ | 3.1 ± 0.1^B^ | 0.0982 ± 0.0013^C^ | 12.26 ± 0.6^C^ | 63.0 ± 1.0^C^ | 36.7±1.5^A^ | 63.3±1.5^A^ | 78.7±1.5^A^ | 84.0±2.0^A^ |
| Δ*Moemc2/MoRGS1*^5D^ | 3.9 ± 0.1^B^ | 3.0 ± 0.1^B^ | 3.2 ± 0.1^B^ | 3.4 ± 0.1^A^ | 0.1070 ± 0.0006^B^ | 31.34 ± 1.0^B^ | 70.7 ± 0.6^B^ | 21.0±1.0^B^ | 45.0±1.0^B^ | 69.3±1.5^B^ | 77.0±1.0^B^ |
| Δ*Moemc2/MoEMC2* | 4.4 ± 0.2^A^ | 3.8 ± 0.1^A^ | 3.5 ± 0.1^A^ | 3.7 ± 0.1^A^ | 0.1280 ± 0.0005^A^ | 59.18 ± 1.2^A^ | 78.7 ± 1.5^A^ | 8.3±0.6^C^ | 19.0±1.0^C^ | 63.7±0.6^C^ | 70.3±1.5^C^ |
